# Novel pH-activatable NIR fluorogenic spray mediated near-instant and precise tumor margins identification in human cancer tissues for surgical resection

**DOI:** 10.7150/thno.85651

**Published:** 2023-08-15

**Authors:** Zhongyuan Xu, Jianqiang Qian, Hongmei Wu, Chi Meng, Qian Ding, Weizhi Tao, Chang-Chun Ling, Jun Chen, Peng Li, Yumin Yang, Yong Ling

**Affiliations:** 1 School of Pharmacy and Jiangsu Province Key Laboratory for Inflammation and Molecular Drug Target, Nantong University, 226001 Nantong, Jiangsu, PR China.; 2 Department of General Surgery, Affiliated Hospital of Nantong University, 226001 Nantong, Jiangsu, PR China.; 3 Department of Hepatobiliary surgery, Nantong Third People's Hospital and the Third Affiliated Hospital of Nantong University, 226001 Nantong, Jiangsu, PR China.; 4 Nantong Key Laboratory of Small Molecular Drug Innovation, Nantong University, 226001 Nantong, Jiangsu, PR China.

**Keywords:** pH-activatable, NIR fluorogenic spray, rapid and precise imaging of clinical tumor tissues, image-guided surgical resection, tumor margin identification

## Abstract

**Rationale:** Challenges such as developing a universal tumor-specific probe for tumor margin identification in diverse tumors with an easy-operative and fast-imaging pattern still exist. Hence, in the present study, a rapidly “off-on” near-infrared (NIR) fluorescent probe **NBD** with pH-activatable fluorescence and a large Stokes shift was constructed for spray mediated near-instant and precise clinical tumor margins identification.

**Methods: NBD** was designed and synthesized by introducing both diphenyl amino group and benzo[e]indolium to β-carboline at C-6 and C-3 positions respectively. The optical properties of **NBD** was characterized by absorption spectra, fluorescence spectra. Subsequently, we investigated its pH-dependent mechanism by ^1^H NMR and density functional theory (DFT) calculation. **NBD** was further under deeper investigation into its imaging performance in nude mice models (subcutaneous, orthotopic, metastatic tumor), and clinical tissues from patients with three clinically representative tumors (liver cancer, colon cancer, and lung cancer).

**Results:** It was found that **NBD** had NIR fluorescence (742 nm), a large Stokes shift (160 nm), and two-photon absorbance (1040 nm). Fluorescence quantum yield (Ф_F_) increased by 5.5-fold when pH decreased from 7.4 to 4.0, to show pH-dependent property. Furthermore, **NBD** could not only selectively light up all four cancer cell lines, but also delineate xenograft tumor and orthotopic microtumor to guide surgical tumor resection, and track metastatic tissues. Particularly, after simple topical spray (three minutes later), **NBD** could rapidly and precisely distinguish the boundary ranges of three kinds of clinical cancer specimens including liver, colon, and lung cancers, with high tumor-to-normal tissue signal ratios (6.48~9.80).

**Conclusions:** Therefore, the proposed fluorescent probe **NBD** may serve as a versatile NIR fluorogenic spray for the near-instant visualization of tumor margins and assisting surgeons in surgerical resection of clinical cancers.

## Introduction

Surgical removal is one of the main and sometimes the only curative options in solid tumor treatment 1,2. Successful surgery is characterized by complete tumor removal and surrounding healthy tissue preservation to minimize cancer recurrence and functional impairment 3,4. Current surgeons demarcate tumor boundaries mainly by palpation and visual inspection, which are insufficient to distinguish malignant tissues from normal tissues during surgery 5,6. Though experienced surgeons can usually achieve better surgical results, tumors such as breast tumors are mostly nonpalpable, consequently causing positive-margin rates in the range of 5%-49% 7. Thus, precisely distinguishing tumors and confirming tumor margin during surgery aid in efficient and accurate tumor resection and reduced tumor recurrence, thereby increasing success rate of surgery and patient survival 8,9. Conventional imaging modalities such as magnetic resonance imaging and computed tomography are suitable only for preoperative planning, not intraoperative guidance. Therefore, fluorescence imaging has been increasingly applied in intraoperative navigation for its unique benefits, including real-time imaging, quick feedback, easy operation, low cost, and safety 10-12.

To date, over 80% of the fluorescent probes used in clinical trials are “always-on” probes, emitting fluorescence regardless of their interactions with target tissues, thus resulting in non-specificity and a low tumor-to-normal tissue ratio (TNR) 13,14. In contrast, stimulus-activatable fluorescent probes are not fluorescent until they are activated by specific tumor biomarker (E.g., overproduced enzymes, low pH) and are promising for *in vivo* imaging 15-17. 5-Aminolevulinic acid (5-ALA) was the only “off-on” probe used clinically, which can elicit the synthesis of fluorescent protoporphyrin IX in cancer cells 18. However, its slow intracellular transformation prohibits the fast-imaging possibility, and a short emission wavelength (634 nm) leads to limited penetration 19. Compared with slow intracellular enzymatic transformation, pH has the potential as a fast and universal stimulus for “off-on” probes. On the other hand, imaging diverse cancers based on specific enzyme catalysis is generally difficult since cancer is genetically or phenotypically heterogeneous 20. Cancer cells are generally characterized by aerobic glycolysis, where glucose is preferentially taken up and subsequently converted into lactic acid to achieve an acidic tumor microenvironment, and this phenomenon is called the Warburg effect 21. The clinical significance of the Warburg effect has been verified by ^18^F-fluorodeoxyglucose utilization in positron emission tomography for imaging non-small cell lung cancer, colorectal, pancreatic, breast, thyroid, melanoma, Hodgkin's and non-Hodgkin's lymphoma, and various types of metastatic cancers to lung, liver, bone, and axillary nodes 22. Therefore, a pH-responsive fluorescent probe is more suitable for broad tumor diagnosis and real-time intraoperative guidance.

Almost all probes are administered via intravenous injection. After intravenous injection, these probes can be transported to the malignant region via blood circulation; however, their metabolism or decomposition during circulation can lead to insufficient accumulation in the tumor site and low availability. Thus, a high dosage of these probes is required via intravenous injection, but it may cause unacceptable system toxicity. Moreover, tumors are characterized by underdeveloped vasculature; hence, probes inside the circulation system have difficulty accessing tiny tumors, resulting in incomplete tumor visualization 23. By comparison, in situ spray and local injection come into our attention since they are fast/easy to perform, and convenient for intraoperative navigation. Furthermore, topical administration allows the probes to avoid circulating metabolism and abate system toxicity in the whole body.

Noteworthily, widely-adopted fluorescent scaffolds such as fluorescein, rhodamine, and cyanine show small Stokes shifts (<30 nm), causing poor TNR and self-quenching 24. Therefore, constructing a novel near-infrared (NIR, >650 nm) fluorescent probe with “off-on” and a large Stokes shift is promising for intraoperative navigation but still challenging. Recently, two-photon imaging has become popular in biological research for its advantageous features over one-photon imaging such as increased penetration depth and reduced photodamage and autofluorescence interference 25-27. However, almost no two-photon fluorescent probes have been used in clinical trials.

**Figure 1:**
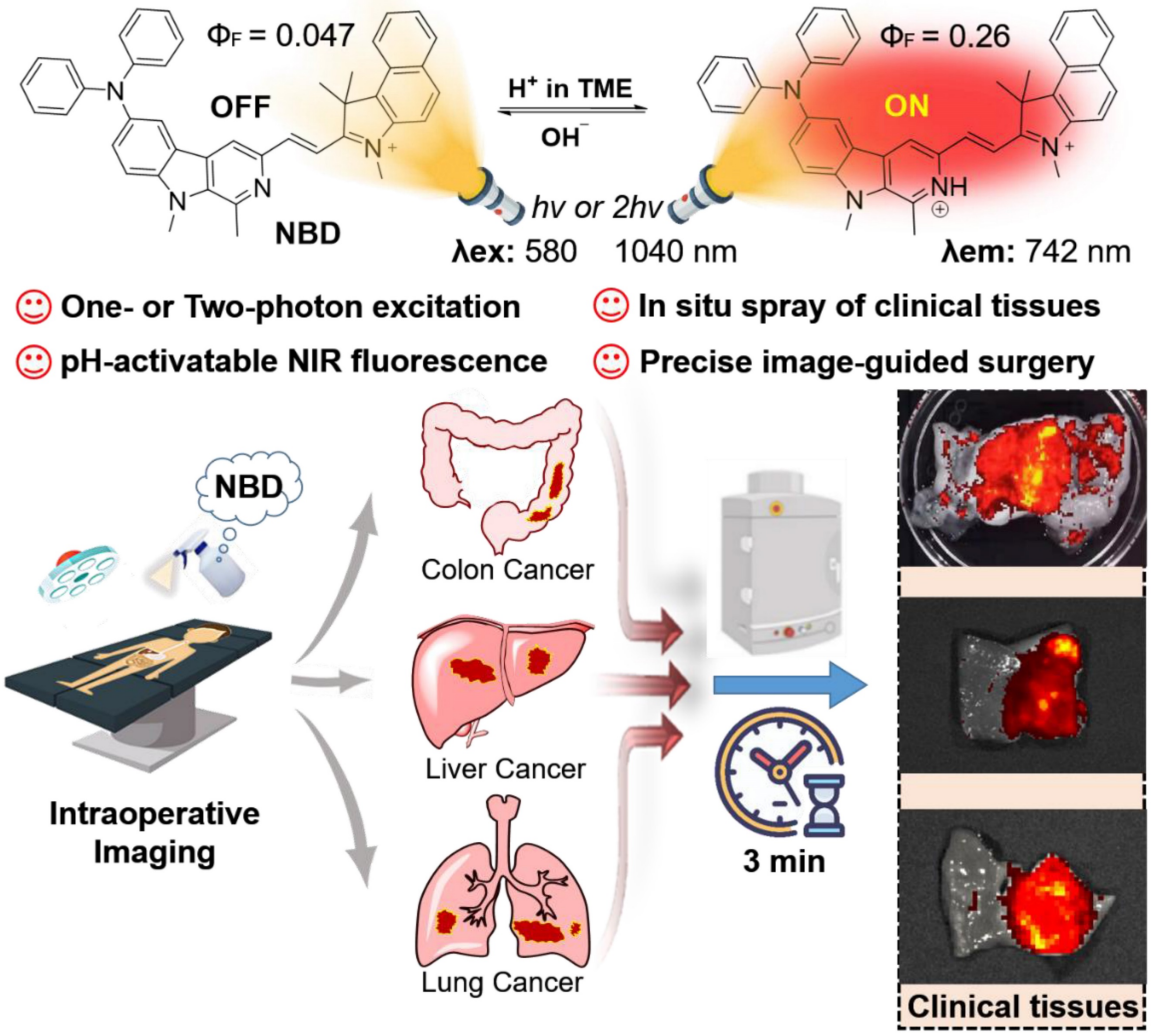
Schematic illustration of pH-activatable NIR probe **NBD** applied to the effective delineation of clinical tumor tissues for image-guided surgery.

β-carboline, a well-known alkaloid derived from plants, shows potential antitumor activities and fluorescence imaging functions 28-32. We are committed to developing the imaging application of β-carboline and first discovered that 3-amino-β-carboline could show pH-sensitive fluorescence to act as a fluorescent scaffold of tumor diagnostic agents 33. However, 3-amino-β-carboline suffered from a short fluorescence wavelength (<510 nm) and a small Stokes shift (<55 nm). In this contribution, electron-rich β-carboline was first conjugated with an electron-withdrawing benzo[e]indolium to build a donor (D)-π-acceptor (A) skeleton, to give bathochromic fluorescence and two-photon absorption (TPA). Furthermore, we introduced diphenyl amino group to C-6 position of β-carboline to increase the electron-donating ability of the donor (β-carboline), hoping to increase intramolecular charge transfer (ICT) to obtain a large Stokes shift and much more bathochromic fluorescence. Hence, a novel fluorescent probe **NBD** was designed and synthesized (**Figure 1**). We expect **NBD** to show “off-on” NIR fluorescence, TPA, and a large Stokes shift simultaneously exhibiting pH-sensitive characteristics so that rapid and selective tumor tissues imaging and diagnosis with high TNR would help surgeons accurately identify and remove tumor tissue. We will validate the above hypothesis by performing a series of *in vitro* and *in vivo* experiments, followed by exploring its application in visualization of tumor margin in clinical tissues.

## Methods

### Cell Culture

Human hepatocellular carcinomas cell line (HepG2), human breast adenocarcinoma cell line (MCF-7), human colorectal adenocarcinoma cell line (HT29), human lung adenocarcinoma cell line (A549), human normal liver cell line (LO2) and human normal lung cell line (HFL-1) were cultured with DMEM medium in 37 °C, 5% CO_2_, atmosphere. DMEM medium was with 10 % fetal bovine serum.

### Intracellular fluorescence by one-photon excitation or two-photon excitation

HepG2 cells, LO2 cells, A549 cells and HFL-1 cells were grown in 35 mm cell culture dish. After cell attachment, these cells were cultured with DMEM medium with **NBD** (2 μM) for 1 h. After that, these cells were washed with PBS three times and imaged under a confocal microscope with excitation wavelength at 582 nm and filter set ranging from 700 nm to 740 nm.

### Mitochondria and lysosome colocalization assay

HepG2 cells, A549 cells, MCF-7 cells and HT29 cells were grown in 35 mm cell culture dish. After cell attachment, the cells were stained with **NBD** (2 µM) for 1 h and then stained with MitoTracker Green and Lysotracker Green. The fluorescence images were obtained using a confocal microscope. **NBD** laser excitation at 582 nm, filter set: 700-740 nm; MitoTracker Green laser excitation at 488 nm, filter set: 515-550 nm; Lysotracker Green laser excitation at 488 nm, filter set: 515-550 nm.

### Animal models

All animal procedures were under the guideline approved by Animal Research and Care Committee (ARCC) of the Nantong University (Approval number: S20210925-003). Female nude mice and Balb/c mice aged 4-5 week were purchased from the Model Animal Research Center Affiliated to Nanjing University (Nanjing, China). Subcutaneous injection of HepG2 cells (1 × 10^6^) in nude mice was operated to establish xenograft tumor models. Injection of A549 cells (8 × 10^5^ cells) into lungs of nude mice was operated and the orthotopic lung tumor models were established after 2 weeks. Intravenous injection of 4T1 cells (2 × 10^6^) in Balb/c mice was operated and metastatic breast cancer models were established after 3 weeks.

### *In vivo* imaging and image-guided surgery

After the tumors reached ~300 mm^3^,** NBD** (40mg/kg) was intratumorally injected into tumor-bearing nude mice. The whole fluorescence imaging was performed on IVIS imaging system at 0, 1, 2, 8, 12, 24, and 48 h postinjection with excitation wavelength at 600 nm.

After HepG2 tumor-bearing nude mouse were anesthetized, the tumor and adjacent normal tissue was carefully exposed with sterile surgical tools. The mouse was imaged using IVIS imaging system under a color scale in advance. Subsequently, **NBD** solution (50 μM, 5% DMSO, and 10% Tween 80 in saline, v/v) were sprayed onto the exposed tumor and surrounding area (the optical properties of **NBD** in the system was shown in **Figure S11**). Three minutes later, the fluorescence imaging of the whole mouse was captured using IVIS imaging system under the initial color scale. Under guidance of imaging, the fluorescent region was resected. Subsequently, **NBD** was sprayed again to ensure that there was nearly no fluorescence in the region, suggesting the complete resection. λ_ex_ = 600 nm.

For image-guided resection of orthotopic lung tumor, the lung of the orthotopic A549 tumor-bearing nude mouse was exposed, under anesthesia. The mouse was imaged using IVIS imaging system under a color scale in advance. **NBD** solution (50 μM, 5% DMSO, and 10% Tween 80 in saline, v/v) was sprayed onto the whole lung. Three minutes later, the fluorescence imaging of the whole mouse was carried out on IVIS Spectrum imaging system under the initial color scale. Under guidance of imaging, the fluorescent region was resected. After that, the mouse was sacrificed and the fluorescence intensity of lung was quantitatively compared with that of the resected tumor. The excised resected lung and tumor were fixed in 4% v/v formalin overnight and embedded in paraffin for histological analysis. λ_ex_ = 600 nm.

### Spray mediated fluorescence imaging in metastatic cancer models

After establishing the metastatic cancer models in nude mice, the nude mice were euthanized and their main organs including heart, liver, spleen, lung, and kidneys were discreetly taken out. The resected organs were imaged using IVIS imaging system under a color scale in advance. **NBD** solution (50 μM, 5% DMSO, and 10% Tween 80 in saline, v/v) was prepared and sprayed onto these organs. Fluorescence images were achieved using IVIS imaging system under initial color scale just three minutes later (λex = 600 nm). The excised organs were fixed in 4% v/v formalin overnight and embedded in paraffin for histological analysis.

### Statistical analysis

Quantitative data were expressed as mean ± standard deviation (SD). Statistical significance was analyzed using a Student's t-test.

## Results

### Synthesis of NBD

**Figure 2:**
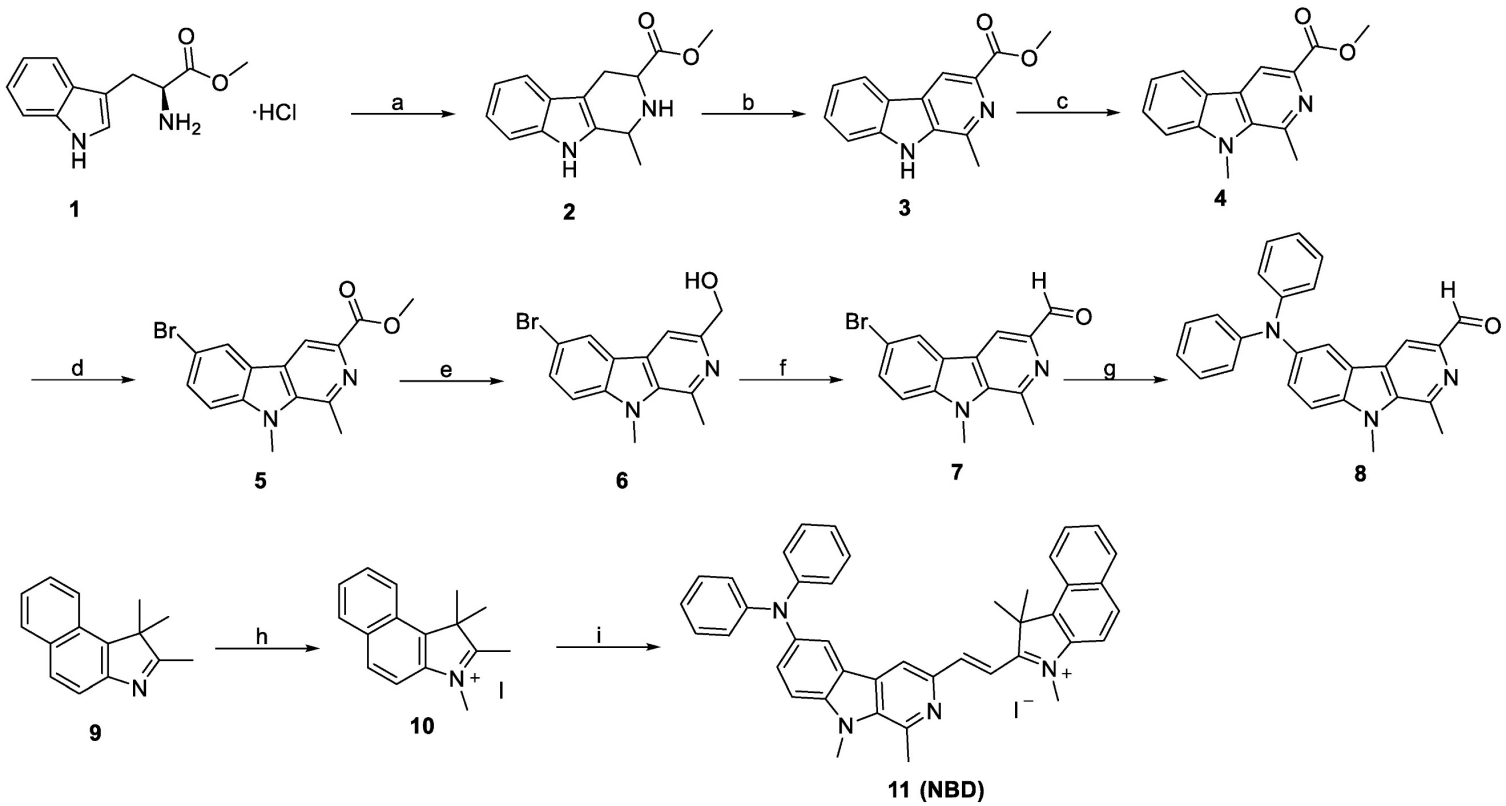
**Synthetic route to NBD**. (a) (i) (C_2_H_5_)_3_N, DCM, rt, 1 h; (ii) CH_3_CHO, r.t., 30 min, and then CF_3_COOH, DCM, r.t., 3 h, 80%; (b) KMnO_4_, DMF, 0 °C, 6 h, 62%; (c) NaH, anhydrous DMF, -5 °C, 30 min, and then CH_3_I, r.t., 3 h, 85%; (d) NBS, TBAB, THF, 45 °C, 1 h, 91%; (e) LiAlH_4_, anhydrous THF, r.t., 3 h, 68%; (f) DMP, DCM, r.t., 2 h, 67 %; (g) diphenylamine, Pd(dba)_2_, sodium tert-butoxide, tri-tert-butylphosphine, toluene, 110 °C, 12 h, 76%; (h) CH_3_I, methanol, 80 °C, 8 h, 98%; (i) **8**, piperidine, ethanol, 80°C, 3 h, 87%.

The designed fluorescent probe **NBD** was synthesized via a nine-step route (**Figure 2**). We selected L-tryptophan methyl ester hydrochloride (**1**) as the starting compound. Compound **2** was first obtained by the Pictet-Spengler reaction and then oxidized using KMnO_4_ to obtain the β-carboline skeleton (**3**). After methylation with methyl iodide, the resulting compound **4** was brominated using N-bromosuccinimide (NBS). Compound **5** was then reduced using LiAlH_4_, followed by Dess-Martin periodinane oxidation to obtain compound **7**. This intermediate **7** was then subjected to Buchwald-Hartwig cross-coupling followed by Knoevenagel condensation with methylated benzo[e]indolium (**10**) to yield **NBD** with a high field. The key intermediates and **NBD** were characterized by ^1^H NMR, ^13^C NMR and high-resolution mass spectrometry (HRMS).

### pH-sensitive optical properties of NBD

**Figure 3:**
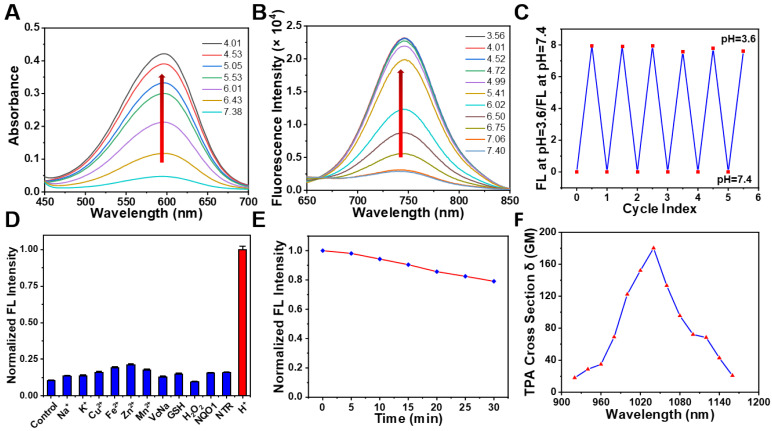
**Optical properties of NBD** (A) Absorption spectra of **NBD** (10 μM) in deionized water (25% v/v methanol) at different pH. (B) Fluorescence intensity of **NBD** in deionized water (25% v/v methanol) at different pH. λ_ex_ = 582 nm. (C) Fluorescence reversibility of **NBD** (10 μM) with pH = 3.6 and 7.4. (D) Fluorescence intensity of **NBD** at 742 nm when treated with various physiologically relevant species or adjusted to pH = 4.0 (mean ± SD, n = 3). (E) Photostability of **NBD** in deionized water under continuous laser irradiation (650 nm, 200 mW/cm^2^) for 30 min. (F) The two-photon absorption cross section at different excitation wavelengths for **NBD**.

To understand whether our designed probe **NBD** could be applied in selective imaging in an acidic environment, absorption and fluorescence spectroscopies were performed under different pH values. **NBD** was first dissolved in methanol to prepare the stock solution (5 mM), which was then diluted using deionized water containing 25% methanol (v/v) to prepare the** NBD** experiment solution. With a decrease in pH, the absorption peak at 582 nm gradually increased (**Figure 3A**). Upon excitation at 582 nm, no clear fluorescence signal was observed from 600 nm to 850 nm at pH = 7.40. However, as the pH decreased from 7.40 to 3.56, a gradually increasing fluorescence emission peak in the NIR region (at 742 nm) was observed, with the maximum value increasing from 2877 to 22941 (~8.0-fold) (**Figure 3B**). The pKa of **NBD** was calculated to be 6.02 (**Figure S1**). Though indocyanine green (ICG) and methylene blue (MB) are widely-used clinical NIR fluorescent probes, they show small Stokes shifts (ICG, 15 nm; MB, 21 nm) 34. Notably, **NBD** showed a large Stokes shift of 160 nm, which may offer better imaging performance with less self-quenching and a high TNR. Furthermore, the fluorescence quantum yield of **NBD** at pH = 7.4 was 0.047, whereas it increased to 0.26 (~ 5.5-fold) at pH = 4.0, thus quantitatively verifying its pH-dependent fluorescence (**Table S1**).

### Photophysical properties of NBD

Considering that pH in a biological environment is dynamic, the reversibility of **NBD** was assessed. As shown in** Figure 3C**, the **NBD** testing solution was modulated back and forth between pH 3.6 and 7.4. The fluorescence intensity ratio at pH=3.6/pH=7.4 was nearly unchanged for more than six cycles, exhibiting remarkable reversibility to pH change.

The selectivity of an activatable probe to stimulus is another key parameter affecting the imaging performance. Therefore, the selectivity of **NBD** to pH over physiologically associated species including crucial metal ions (Na^+^, K^+^, Cu^2+^, Fe^2+^, Zn^2+^, and Mn^2+^), small bioactive molecules (glutathione and vitamin C), reactive oxygen species (H_2_O_2_), and enzymes (NAD(P)H quinone oxidoreductase 1 [NQO1] and Nitroreductase [NTR]) was investigated. As expected, besides H^+^, negligible fluorescence signals were observed after adding other physiologically associated species, indicating the great specificity of **NBD** to pH (**Figure 3D**).

In order to assess the photostability of **NBD**, **NBD** was exposed to light (650 nm, 200 mW/cm^2^). As a result, there was a slight drop (20.9%) in terms of **NBD** fluorescence after continuous light exposure for 30 min (**Figure 3E**). In comparison, fluorescence intensity of ICG dramatically dropped by > 90% within 30 min 35. These results suggest **NBD** exhibited acceptable photostability. Coupled with NIR fluorescence and a large Stokes shift, high reversibility selectivity, and photostability offered **NBD** a great potential in biological applications.

Two-photon imaging is superior to conventional one-photon imaging because of its minimal interference from background species, high imaging depth, and low photodamage 23-25. The potential of **NBD** in two-photon imaging was thus evaluated. **Figure 3F** shows the maximum two-photon absorption cross section of 180 GM at 1040 nm. The two-photon excitation in the second NIR window (NIR-II, 1000-1700 nm) may lead to deeper penetration and a higher signal-to-noise ratio.

### Density functional theory (DFT) calculation

**Figure 4:**
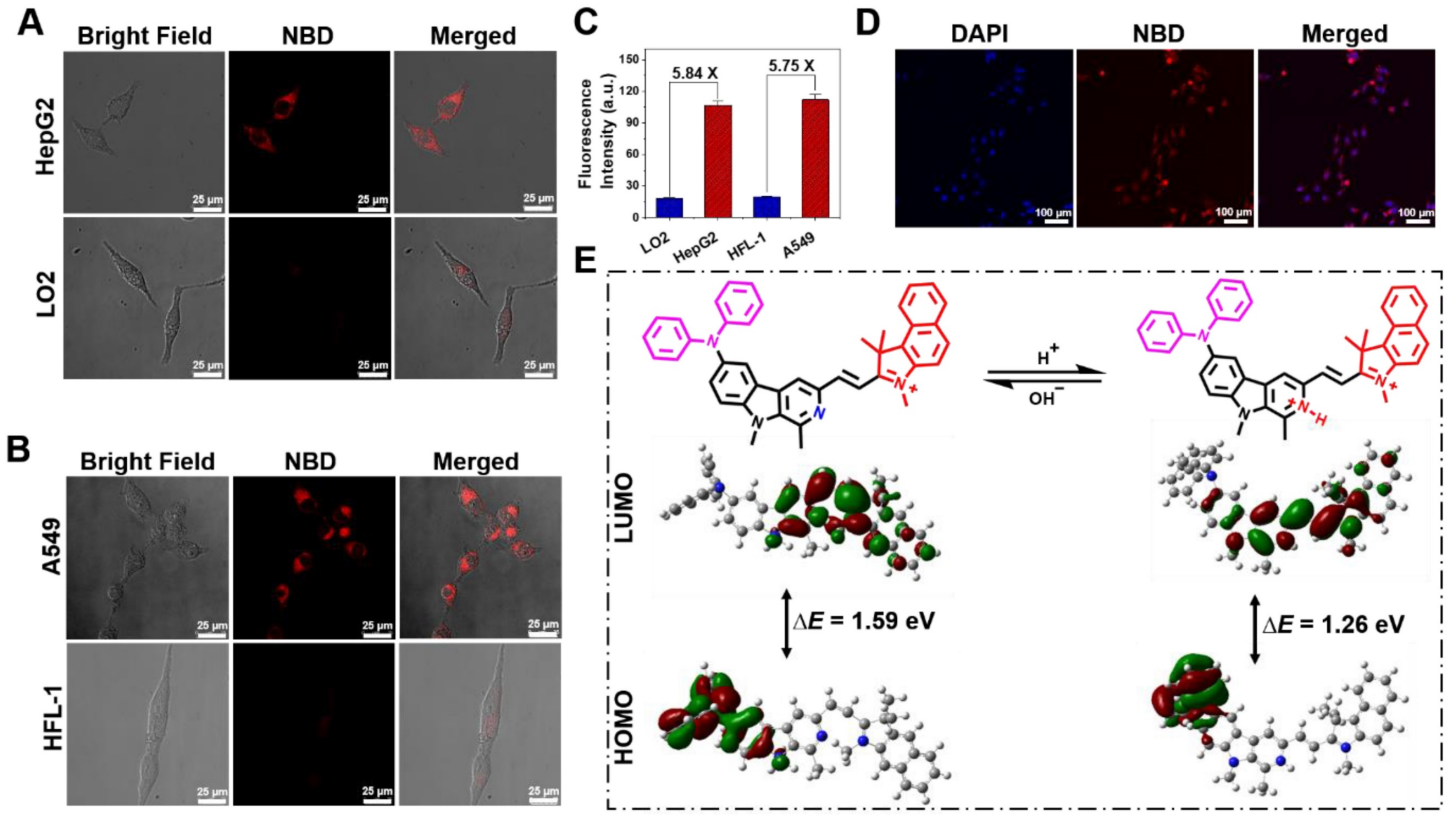
**Cell imaging and DFT calculation.** (A,B) Fluorescence images of liver cancer cell line (HepG2), lung cancer cell line (A549), normal liver cell line (LO2), and lung fibroblast cell line (HFL-1) incubated with **NBD** by one-photon excitation (λ_ex_ = 600 nm). Scar bar = 25 μm. (C) The average fluorescence intensity of different cells incubated with** NBD** for 1 h (mean ± SD, n = 3). (D) Two-photon fluorescent confocal image of HepG2 cells stained with **NBD** (λ_ex_ = 1040 nm). Scale bar = 100 μm. (E) The frontier molecular orbitals (FMOs) involved in **NBD** with deprotonated and protonated pyridine based on DFT calculations using the Gaussian 09 software.

According to our hypothesis, the pH-dependent feature of **NBD** may be attributed to the changed ICT effect originating from the protonation of the β-carboline unit in an acidic environment.^ 1^H NMR and ^1^H-^1^H COSY NMR spectra of **NBD** at pH = 7.4 and 4.0 were examined and the chemical shift of key Hydrogen atom in proximity to three nitrogen was monitored. As shown in **Figure S2-S5,** under neutralized condition (pH=7.4), NMR signal of hydrogen atom *a*, *b*, *c*, *d*, *e*, *f*, *g* was observed at δ ~7.02, ~7.28, ~7.10, ~7.98, ~8.42, ~3.17, ~4.23 ppm, respectively. The addition of deuterated TFA could induce the down-field shift for above hydrogen signal. Among these, *e* and *f* present the most two outstanding NMR signal change (Δδ[*e*] = 0.63 ppm, Δδ[*f*] = 0.29 ppm). This indicates protonation mainly occurs on Nitrogen of pyridine at pH=4.0 to show the deshielding effect on the nearby Hydrogen signal (*e*, *f*). Therefore, we deduced that protonation mainly occurs on the Nitrogen of pyridine when **NBD** maintains at acidic tumor microenvironment. To better understand the molecular mechanism and optical changes in **NBD** upon protonation, DFT calculations were performed in deprotonated/protonated states. Briefly, the optimized structures, the highest occupied molecular orbital (HOMO), and the lowest unoccupied molecular orbital (LUMO) of deprotonated/protonated **NBD** were analyzed and are presented in **Figure 4E**. According to the optimized structures and calculated frontier molecular orbitals, the HOMO was mainly localized on the diphenylamine unit, whereas the LUMO spread over the carboline, olefinic bond, and benzindole, suggesting an obvious ICT from diphenylamine to nitrogen-containing heteroaromatic rings and the olefinic bond upon excitation. Compared with the HOMO-LUMO energy gap (Δ*E* = 1.59 eV) of the deprotonated form, protonated **NBD** had a narrow energy gap (1.26 eV), leading to the bathochromic shift of the absorption and enhanced emission spectra.

### Selective fluorescence imaging of NBD in cancer cells

Given that acidic pH is a universal marker of TME 36, we surmised pH-dependent **NBD** could be used to distinguish cancer cells from normal cells. In order to verify our assumption, we investigated the fluorescence of **NBD** in liver and lung cancer cells as well as related normal cells. Prior to cell imaging studies, the toxic effects of **NBD** on normal cell line (LO2) was examined to evaluate its biocompatibility which is considered as a prerequisite to cell and *in vivo* imaging. The cell viability results presented in **Figure S6** showed that **NBD** had low toxicity with cell viability > 95% at its working concentration and > 80% even at 50 μM, thus suitable for biological imaging.

The fluorescence performances of **NBD** in a liver cancer cell line (HepG2), lung cancer cell line (A549), normal liver cell line (LO2), and lung fibroblast cell line (HFL-1) were evaluated by confocal laser scanning microscopy. After incubation with **NBD**, obvious fluorescence signals were observed in the cancer cell lines, whereas the normal cell lines showed nearly no intracellular fluorescence (**Figure 4A** and **B**). As shown in **Figure 4C**, the quantifications of intracellular fluorescence showed that fluorescence intensities in the cancer cell lines were over 5.7-fold higher than those in two normal cell lines, indicating the selective imaging of **NBD** in cancer cells.

In view of the maximal two-photon absorption cross section at the NIR-II region, we used the femtosecond two-photon excited fluorescence technique to evaluate the two-photon fluorescence of **NBD** in living cells. As shown in **Figure 4D**, after incubation with HepG2 for 30 min, **NBD** showed bright fluorescence in the cancer cells under two-photon excitation at 1040 nm, indicating its capability of image living cancer cells under two-photon excitation.

### Selectively targeting mitochondria in cancer cells

**Figure 5:**
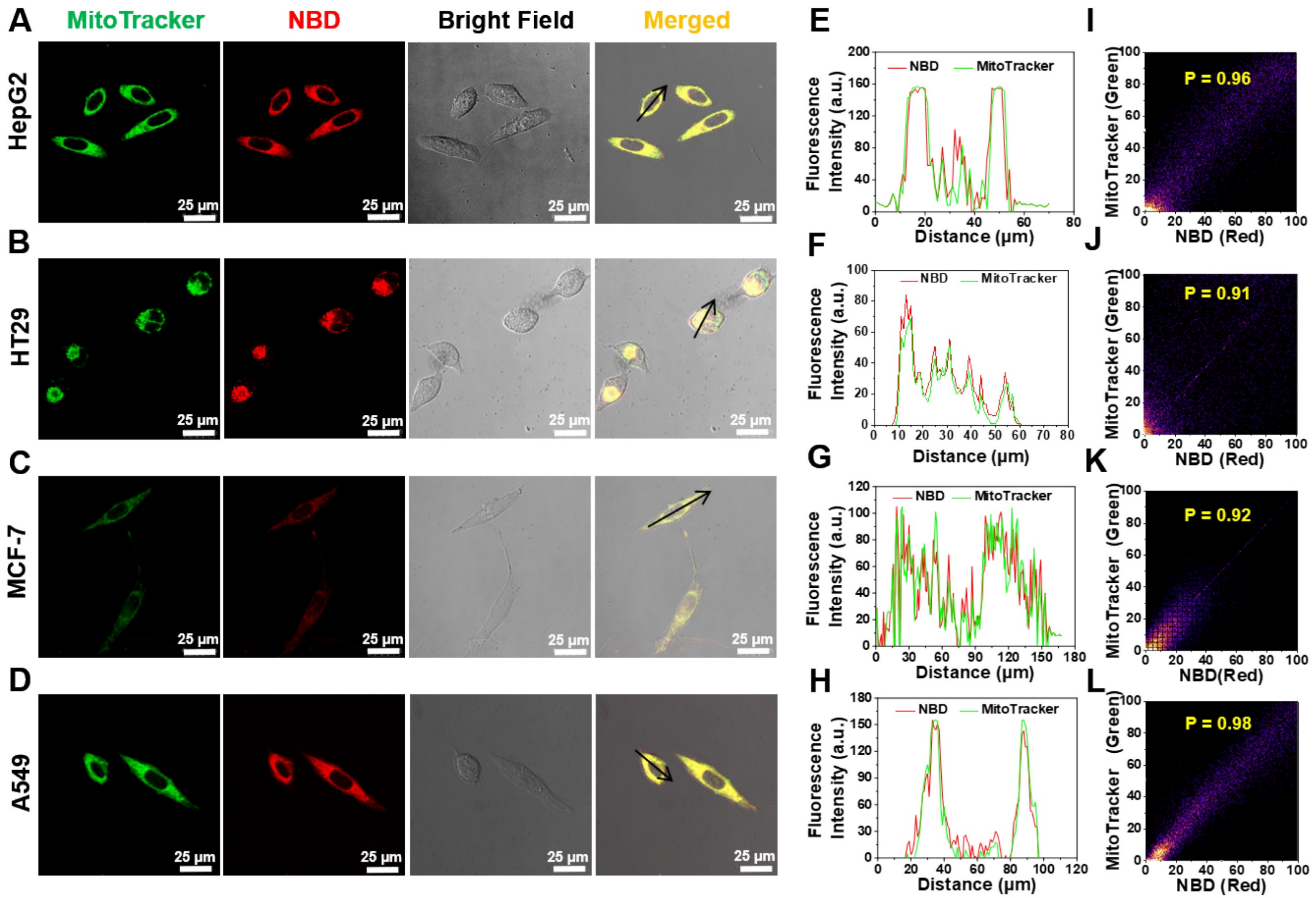
** Intracellular mitochondrion colocalization.** (A-D) HT29 (A), HT29 (B), MCF-7 (C), A549 (D) cells were pretreated with **NBD** for 1 h and then incubated with MitoTracker Green for 30 min. (E-H): The intensity profiles along with the black arrow of cancer cells from (A)-(D). (I-L): Plots representing the intensity correlation of MitoTracker Green and **NBD**. **NBD** was excited at 600 nm. MitoTracker Green were excited at 488 nm. Scale bar = 25 μm.

Considering that **NBD**-containing lipophilic cationic unit can tend to accumulate in mitochondria in living cells, the intracellular colocalization of **NBD** was then investigated 37-39. HepG2, breast cancer cell line (MCF-7), HT29, and A549 were co-stained with **NBD** and a commercially available organelle probe (Lysotracker Green as a lysosome probe or MitoTracker Green as a mitochondrial probe). As shown in **Figure 5A-D**, after successive incubation with **NBD** and MitoTracker Green, all four tested cancer cell lines emitted intense fluorescence (visualized as red) and overlapped well with the green fluorescence of MitoTracker to show yellow signals, and all Pearson's coefficients were over 0.90 (**Figure 5I-L**). Higher magnification cell images were obtained and displayed in **Figure S8,** wherein Pearson's coefficient was up to 0.93. In contrast, the fluorescence of Lysotracker Green did not match well with the fluorescence of **NBD** according to lower Pearson's coefficients (**Figure S7**). These results showed that **NBD** was mainly accumulated in the mitochondria of the cancer cells after cellular uptake.

### Intraoperative image-guided surgery in xenograft tumor models

**Figure 6:**
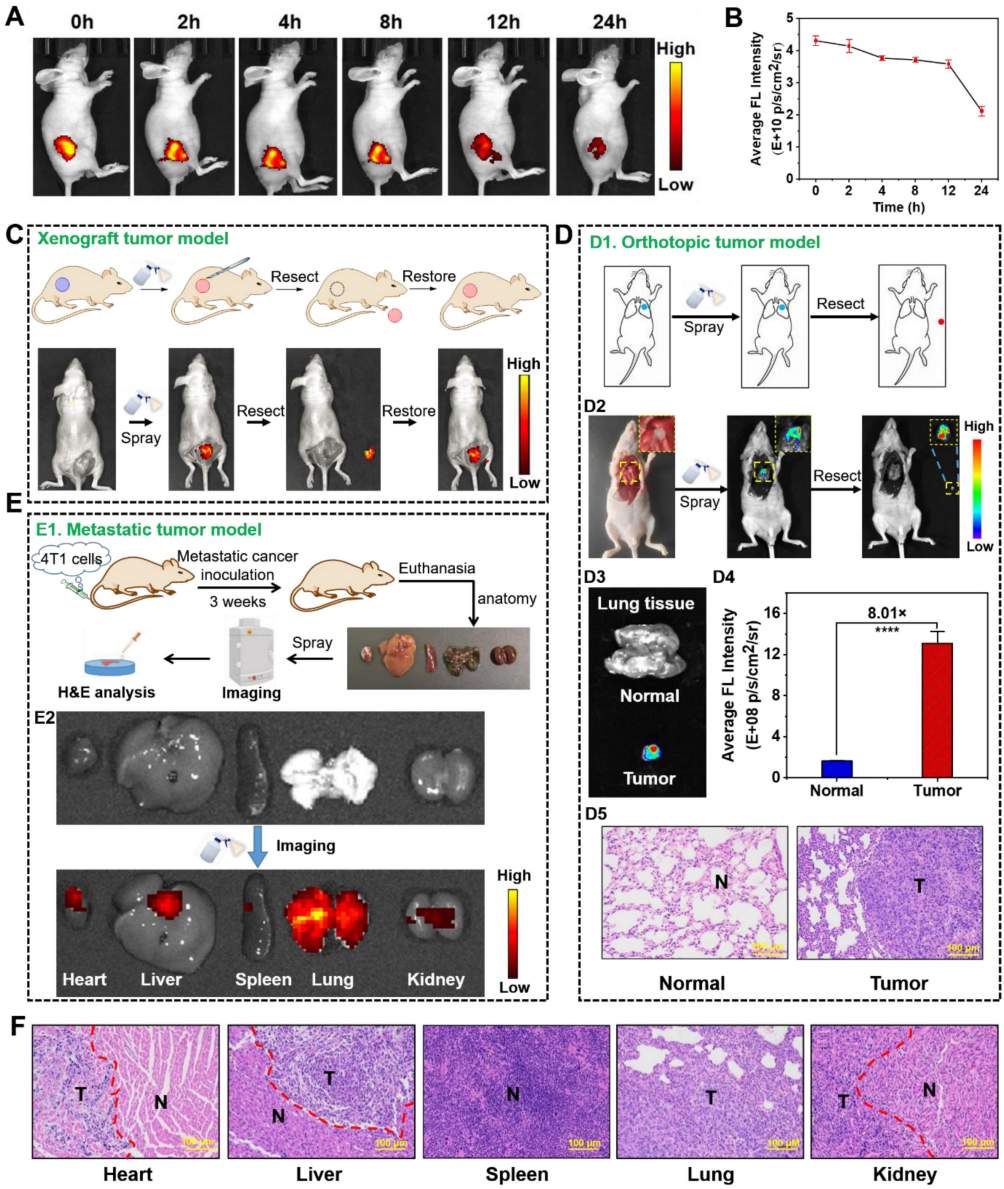
***In vivo* fluorescence visualization and image-guided surgery in tumor-bearing nude mice.** (A) Fluorescence imaging of tumor-bearing mice at different time-points after an intra-tumoral injection with **NBD** (40 mg/kg). (B) Average fluorescence intensity of the tumor region in the xenograft tumor nude mice at different time points after injection (mean ± SD, n = 3). (C) The procedure of fluorescence-guided surgery by *in situ* spraying **NBD** in xenograft tumor models. (D): (D1) Schematic illustration of fluorescence-guided surgery through spraying in orthotopic tumor models; (D2) fluorescence-guided surgery of orthotopic A549-tumors using **NBD**; (D3) fluorescence images of the excised tumors and normal lung tissue; (D4) the relative average fluorescence intensity of (D3); (D5) H&E staining of the resected tissue and normal lung tissue. (E): (E1) Schematic illustration of *ex vivo* imaging of main organs from 4T1 metastasis-bearing models; (E2) fluorescence imaging of the excised lung metastases and organs after spraying **NBD**. (F) H&E staining of a section of the organs in the metastasis-bearing model. T: tumor tissue, N: normal tissue, scale bar = 100 μm.

Encouraged by the selective NIR fluorescence of **NBD** in the cancer cells, we assessed its imaging performance in tumor-bearing models. **NBD** was injected intratumorally into nude mice with transplanted tumors, and fluorescence intensity in the tumor area was monitored on Caliper IVIS Lumina II optical imaging system at different time points post-injection (0, 2, 4, 8, 12, and 24 h). An intense NIR fluorescence signal was observed upon the excitation at 600 nm (within 3 min). As time passed, the fluorescence faded away but could be visualized even after 24 h, suggesting the rapid imaging property and long tumor retention ability of **NBD** (**Figures 6A** and** 6B**).

As discussed earlier, NIR fluorescence-guided surgery using molecular probes has attracted huge attention; however, FDA-approved fluorescent probes have some shortcomings. Given the selective and rapid fluorescence of** NBD** in tumor tissues, we simulated the process of clinical surgery and tested the effectiveness of **NBD**-guided surgery. In detail, the epidermis around the tumor of a tumor-bearing nude mouse was carefully uncovered to disclose the tumor mass and adjacent normal tissues, and **NBD** (50 μM) was then sprayed. Three minutes later, there was obvious fluorescence present in the tumor area, whereas negligible fluorescence was observed in the surrounding area. Under the guidance of the fluorescence, the tumor tissue was resected until the fluorescence signal from the mouse was nearly invisible. Subsequently, **NBD** was sprayed onto the tumor bed and there was no fluorescence observed, indicating that the tumor was removed (**Figure 6C**).

To distinctly assess the specificity of **NBD** to tumors using spray mode, we directly sprayed **NBD** onto excised normal organs and the tumor, followed by evaluating images collected. In detail, other tumor-bearing nude mice were euthanized, and the main organs (the heart, liver, spleen, lung, and kidneys) and tumors were isolated and pre-imaged using IVIS. Subsequently, **NBD** (50 μM) was sprayed onto all excised tissues. As shown in **Figure S12** and** S13**, whole NIR fluorescent images revealed that the tumor yielded a stronger NIR fluorescent signal than any other organ with TNR > 5.1 after 3 min of spraying. The above results confirmed that spraying **NBD** facilitated tumor tissue visualization with high selectivity, highlighting its great potential in image-guided surgery.

We investigated two-photon excited fluorescence imaging in living tumor tissues, where **NBD** could show deep imaging penetration of two-photon imaging up to 237 μm (**Figure S14**). The result suggest that **NBD** has a noteworthy potential for *in vivo* two-photon imaging applications, thus deserving further investigation.

### Intraoperative image-guided surgery in orthotopic tumor models

We further investigated whether **NBD** could be applied to intraoperative orthotopic tumor imaging by imitating clinical surgery. After establishment of orthotopic lung tumor models, the thoracic cavity of the models was surgically exposed and pre-imaged (**Figure 6D1**). **NBD** was directly sprayed onto the thoracic cavity, and then imaged within 3 min. As shown in **Figure 6D2**, the NIR signals indicated the tumor location, which was thus resected. Furthermore, there was an 8-fold higher signal in the resected tumor tissue than the background of the normal lung tissue, and the resected tissue was expectably verified to be tumor tissues by H&E staining, signifying that the tumor was successfully removed under the guidance of **NBD** (**Figure 6D3-D5**). Noteworthy, the resected tumor was as small as 2 mm, which indicated that **NBD** possessed excellent sensitivity to detect microtumors. These data revealed that **NBD** held the ability to guide surgical resection of orthotopic microtumors.

### Imaging and tracking in metastasis-bearing models

Considering that over 90% of deaths from cancer are attributed to metastatic cancers 40, we investigated the ability of **NBD** to rapidly detect metastatic cancers by *in situ* spray (**Figure 6E1**). Therefore, we constructed a metastasis-bearing model in nude mice by intravenously injecting 4T1 cells. After establishment of metastasis-bearing model, the metastasis-bearing mice were sacrificed to collect the main organs (heart, liver, spleen, lung, and kidneys). As clearly shown in the photograph of **Figure S15**, diffuse metastatic tumors covered the lung and a few metastatic nodules on the heart were visible by naked eyes. To investigate whether **NBD** could visualize these metastases, the main organs were pre-imaged using IVIS with a color scale. **NBD** was then uniformly sprayed onto these main organs. *Ex vivo* fluorescence imaging was performed using the same color scale three minutes later. The captured images showed NIR fluorescence spreading over the lung tissues, revealing its ability to visualize the metastasis. It was of note that other organs (liver and kidneys) also showed an extent of fluorescent signals, which might indicate 4T1 cells metastasized to other organs in addition to lung and heart (**Figure 6E2** and** S16**). To verify our hypothesis, these organs were prepared for histological assessment. As expected, the H&E analysis in **Figure 6F** revealed the existence of large-scale metastasis to the lung and partially metastatic tumors in liver, kidney, and heart tissues. It was of note that metastatic lesions on the heart was as small as 2 mm and could also be clearly imaged. These results may indicate that micro-metastatic lesions could be found and removed with the assistance of **NBD** during surgery. The consistent fluorescence imaging and histological analysis results suggested that visualizing and tracking metastatic tumors were possible using** NBD**.

### Precise and rapid imaging of clinical specimens

As mentioned above, there is currently a lack of ways of enabling rapid and accurate tumor boundaries detection in clinical practice. In order to further study the imaging performance of **NBD** on clinical tumor tissues, we selected three clinically representative tumors (liver cancer, colon cancer, and lung cancer) with high incidence to assess ability of **NBD** to recognize tumor tissue and corresponding TNR. Primarily, dosage screen assay was performed to select optimal dosage employed to obtain the highest signal contrast between the tumor and the normal tissue. The clinical cancer tissues and normal tissues were pre-imaged using IVIS with a color scale. Subsequently, **NBD** at different concentrations (0, 10, 20, 50, and 100 μM) was sprayed five times (625 μL) onto the cancer tissue and normal tissue. Three minutes later, the fluorescence images were captured using the initial color scale (**NBD** at 0 μM) to avoid interference from tissue background, followed by assessment of corresponding TNR in different groups. As shown in **Figure S17**, TNR rose as concentration increased, and reached at most 6.32 at 50 μM. Despite the phenomenon that fluorescence in cancer tissue became increased at 100 μM, TNR fell slightly. Therefore, 50 μM was used as the optimized concentration for the application investigation.

Subsequently, we evaluated imaging specificity (TNR) of **NBD** at different tumor types. Three minutes after **NBD** (50 μM) was sprayed onto both normal and tumor tissues, intense fluorescence was generated in liver cancer and colon cancer tissues, while the fluorescence was almost invisible in normal tissues (**Figure S18B** and** S19B**). Moreover, TNR of fluorescence in liver cancer and colon cancer tissues could reach 6.48 and 8.41 respectively (**Figure S18C** and **S19C**). This result fully demonstrated the highly selective fluorescence imaging capability of **NBD** for clinical tumor tissue.

**Figure 7:**
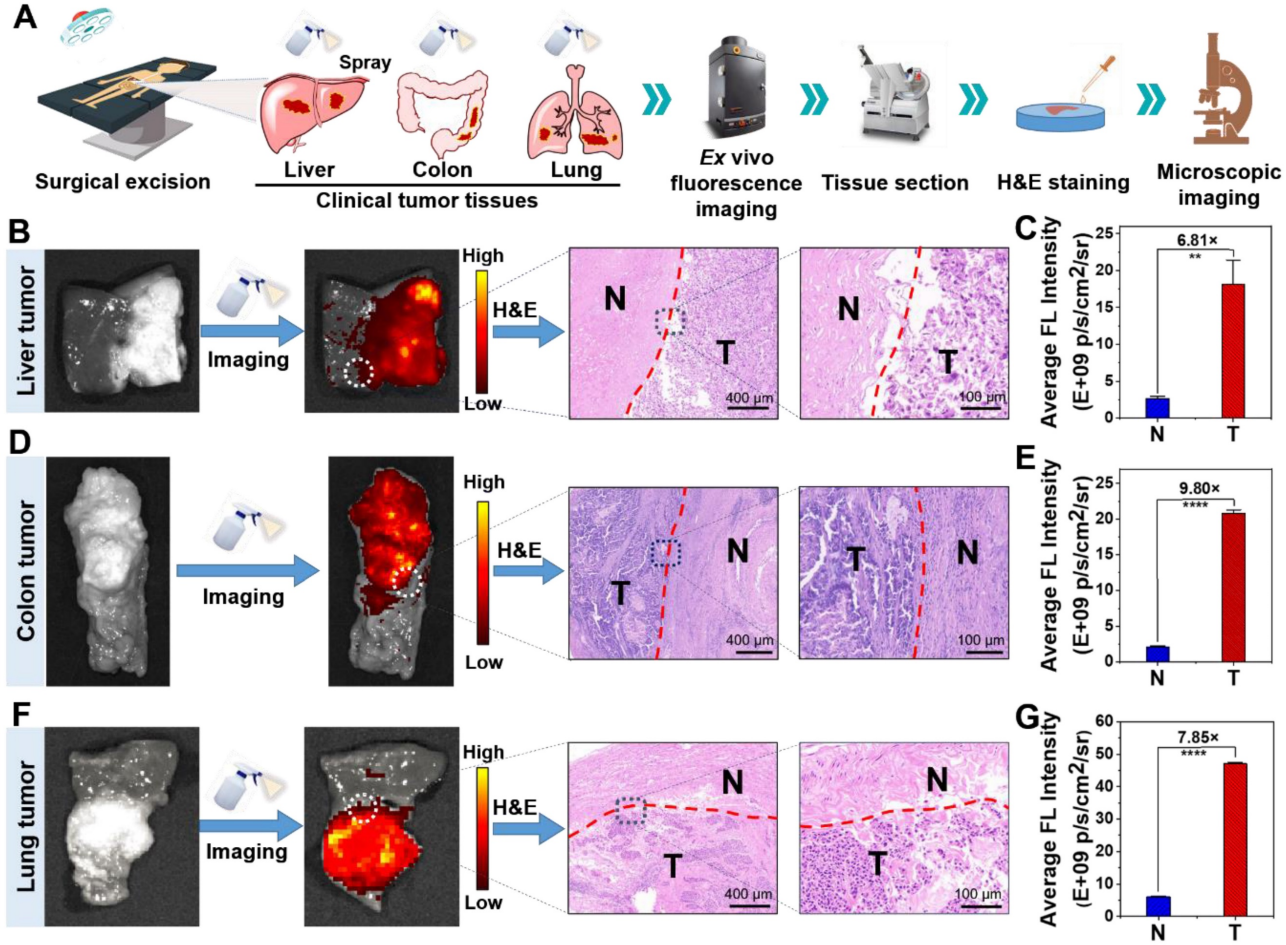
***Ex vivo* spray for clinical cancer identification.** (A) Schematic illustration of cancer detection in human specimens. (B)(D)(F) Representative fluorescence images of the clinical liver (B), colon (D), lung (F) tissues after spraying with **NBD** (50 μM) and H&E analysis of the fluorescence boundary. (C)(E)(G) Quantification of the fluorescence intensity according to panel (B)(D)(F). (**, P<0.01; ****, P<0.0001).

Next, we exploited clinical specimens simultaneously containing both tumor tissue and normal tissue to investigate whether **NBD** spray could be employed to delineate clinical tumor tissue boundaries. In order to facilitate experimental observation, clinical tissue specimens with relatively clear tumor boundaries estimated by visual judgment and tissue hardness were selected to be sprayed with **NBD** (50 μM), and held for 3 min away from light. The captured fluorescence images of the whole tissues showed that significant fluorescence was observed in some areas of the clinical liver cancer (**Figure 7B** and **S18E**), colon cancer (**Figure 7D** and **S19E**), and lung cancer (**Figure 7F** and **S20**) tissue specimens. The fluorescence regions were in good agreement with the suspected cancer tissues by visual inspection, simultaneously with clear demarcation from nonfluorescent tissues. The detailed imaging procedure and results are presented in **Video S1** and **S2**. Further H&E analysis revealed that fluorescence boundary was in line with the tumor boundary indicated by H&E staining (**Figure 7B**,** 7D**, and **7F**). Fluorescence quantization results showed that **NBD** showed high TNR in all three kinds of tumor tissues, among which, the TNR in colon cancer tissues was the highest (TNR = 9.80), and TNR in liver cancer and lung cancer tissues reached 6.81 and 7.85 respectively (**Figure 7C**,** 7E**, and **7G**). These results suggest that **NBD** spray can enable near-instant and accurate tumor margin identification in above three kinds of clinical tumor tissue with high incidence, highlighting the universality and versatility of NIR fluorogenic spray **NBD** in the clinical practice. Taken together, these results indicated that** NBD** spray was capable of imaging tumor tissue selectively and attaining rapid and accurate tumor boundaries detection in clinical practice.

### Biosafety of NBD

The biosafety of **NBD** in mice was investigated by H&E staining and blood analysis. Mice were euthanized at 24 h after intravenous injection of **NBD** for blood/organs collection. Since the dosage corresponding to spraying was 50 μM (about 625 μL), the **NBD** dosage was 1.1 mg/kg which was similar to the spraying dosage. As shown in **Figure S22**, there were no significant histopathological abnormalities or lesions in the **NBD**-treated groups compared with the control group. Whole blood routine examination and serum biochemical determination including aspartate transaminase (AST), alanine transaminase (ALT), alkaline phosphatase (ALP), blood urea nitrogen (BUN), and creatinine (CREA) were also performed. Compared with the control group, there was no significant difference in the whole blood routine (**Figure S23A**). Liver function indexes (ALT, AST, ALP) and renal function indexes (CREA, BUN) were also normal (**Figure S23B**). H&E staining and blood analysis results demonstrated that the **NBD** treatment induced no obvious systemic toxicity, and hepatorenal function impairment.

## Discussion

ICG and MB with NIR fluorescence are representative probes that have been used in clinics 34,35. However, their clinical implementation remains limited to the detection of liver cancers and vascular perfusion assessment. A key reason for this limitation is its non-specificity caused by its “always on” property 13,41. In spite of 5-ALA as an “off-on” probe exhibiting selective fluorescence in cancer cells, emission peak around 630 nm outside the NIR region and slow intracellular enzymatic transformation limited its application in real-time intraoperative guidance 18,19. By contrast, probes with pH-dependent fluorescence are characterized by highly sensitive, fast, and reversible imaging performance and are suitable for detecting many tumors types according to the principle of aerobic glycolysis in TME 42-45.

In the present study, pH-sensitive β-carboline was introduced with diphenyl amino group and benzo[e]indolium at C-6 and C-3 positions, respectively. The novel fluorescent probe **NBD** was designed and synthesized, and exhibited NIR fluorescence (742 nm), a large Stokes shift (160 nm), simultaneously with maintaining the pH-dependent property. **NBD** with the D-π-A skeleton was supposed to exhibit TPA, which was subsequently evaluated. To our surprise, the maximum two-photon cross-section of **NBD** is localized at the NIR-II window (1040 nm), showing immense potential for *in vivo* applications. We discovered the fluorescence of **NBD** enabled two-photon deep tissue imaging with a penetration depth of as much as 237 μm. However,* in vivo* imaging in this research was obtained only by one-photon excitation due to shortage of two-photon imaging equipment. Therefore, its two-photon imaging application needs further exploration.

Recently, Chen et.al. designed a tumor-targeting small molecular probe with both large Stokes shift (158 nm) and NIR emission (650 nm); however, it suffered from non-negligible background interference (TNR < 3) that was attributed to its “always on” fluorescence 46. CLSM images showed that **NBD** selectively fluoresced in all tested four cancer cell types. The specificity of **NBD** to cancer was assessed in the tumor-bearing nude mouse model. Spraying is a promising and easy-to-use method for cancer detection, in avoidance of circulating metabolism. Only 3 min after spraying **NBD**, we observed obvious fluorescence from the resected tumor with a high TNR > 5 (**Figure S13**), which exemplified the superiority of integration of “off-on” fluorescence and large Stokes shift (**Figure S21**). In detail, “off-on” property allowed for accurate imaging of tissues, and large Stokes shift alleviated crosstalk between absorption and emission spectra, minimized self-quenching caused by reabsorption, and enhanced signal readout in tumor site.

Almost all reported or clinically used probes in intraoperative guidance such as Aminopeptidase N-activatable YH-APN (30 min) and “always-on” ICG (14 days) require more time to achieve adequate TNR 47, decreasing their practicality for routine or real-time clinical use, while **NBD** mediated image occurred within 3 min of topically spraying the tumor not only in animals but also in clinical tissues (**video S1** and **S2**). CLSM and flow cytometry proved that **NBD** could rapidly internalize and light up the tumor cells within 3 min (**Figure S9** and **S10**). Additionally, **NBD** spray enabled highly selective tumor diagnosis of both large-scale metastases and small-sized metastatic lesions in the metastatic tumor-bearing model as well. Noteworthily, the lower dose was required for topical administration via spraying (**NBD**, 31 nmol for one mouse) compared to systemic intravenous administration (e.g., ICG, 250 nmol for one mouse) 48, which likely reduced potential toxicity concerns.

To evaluate the clinical versatility and translational potential of **NBD**, its capability of delineating tumor borders from benign tissues was investigated in three common solid tumor types (liver, colon, and lung cancers) with high morbidity (>22.1% in 2020) 49. We confirmed that the **NBD** spray enabled accurate and rapid differentiation of cancer tissues/normal tissues in clinical specimens from patients with liver, colon, or lung cancer, and visualization of the cancer margin within three minutes. The versatility and universality of the** NBD** spray in clinical multiple solid tumors may also be attributed to acid TME that results from excessed aerobic glycolysis, a ubiquitous consequence of cancer cell proliferation, growth, and metastasis. Intraoperative histopathological analysis such as H&E staining is the gold standard for tumor margin determination, but its time-consuming drawback delays intraoperative decision-making. In view of **NBD**'s capability of fast and accurate margin identification, **NBD** spray may serve as a novel alternative method for near-instant specimen analysis. Moreover, **NBD** may be *in situ* sprayed onto the any suspected site directly during the surgery to delineate the borders of tumors and determine whether the residual tumor exist. These applications may realize image-guided surgery and assist surgeons in performing surgical resection. Though Onoyama et.al. applied their probe in clinical esophageal squamous cell carcinoma tissues, the probe needed ~10 min waiting time and was still trapped in the short fluorescence wavelength (524 nm) and small Stokes shift (23 nm) 50. The pH-activatable fluorescent probe reported by Xiong et al. could also rapidly visualize pulmonary and abdominal metastatic tumors within 30s, but they didn't explore the imaging performance and the time necessary in clinical tissues 51. It was thus believed that **NBD** may feature the rapidest imaging performance among all spraying probes applied in clinical tissues (**Table S2**), which further underlined the priority of **NBD** in clinical applications. Based on these results and discussions, **NBD** can be a probe candidate for clinical translation and own a universal application in over 20% of clinical tumor surgery scenarios. Recent analysis demonstrated that each 10% increase in optimal cytoreduction was associated with a 5.5% increase in median survival 52, suggesting **NBD** mediated surgical cytoreduction may be highly beneficial to patient survival enhancement. We intend to expand the scope of tumor types and are exploring the intraoperative imaging application in more solid tumors including breast cancer, pancreatic cancer, carcinoma of the bile duct, cervical cancer, etc. We believe that our study can lay a concrete foundation for the application of this probe and its commercial use in the clinical setting.

## Conclusion

In summary, the “off-on” NIR fluorescent probe **NBD** was designed and synthesized by introducing both diphenyl amino group and benzo[e]indolium to β-carboline at C-6 and C-3 positions respectively.** NBD** not only maintained pH-sensitive fluorescence but also enabled bathochromic fluorescence in the NIR region (742 nm) and a large Stokes shift (160 nm) by enhancing the ICT effect. Quantatively, Ф_F_ increased by 5.5-fold from pH 7.4 to 4.0. Furthermore, **NBD** showed a two-photon absorption at the NIR-II window (1040 nm). Considering these significant optical features, **NBD** selectively illuminated four cancer cell lines with high signal-to-noise ratios. *In vivo* experiments involving in situ spraying proved that **NBD** could delineate xenograft tumors and orthotopic microtumors, and trace metastatic tumors with high TNR and guide successful surgical resection of the tumor in a tumor-bearing mouse. Importantly, spraying **NBD** could be employed to identify the cancer margin between cancer and normal tissues in fresh human clinical cancer tissues of three common cancer types (liver, colon, and lung cancers) within three minutes. Conclusively, our results demonstrated that **NBD** could enable near-instant and precise tumor margin identification, thus manifesting its potential as a versatile imaging tool for intraoperative primary/metastatic tumor monitoring and fast-image-guided surgery.

## Supplementary Material

Supplementary figures and tables.

Supplementary video S1 (Liver).

Supplementary video S2 (Lung).

## Abbreviations

A549: human lung adenocarcinoma cell line
CLSM: confocal laser scanning microscope
DCM: dichloromethane
DMEM: dulbecco's modified eagle medium
DMP: Dess-Martin Periodinane
DMF: N,N-Dimethylformamide
DFT: density functional theory
HFL-1: human normal lung cell line
HepG2: human hepatocellular carcinomas cell line
ICT: intramolecular charge transfer
HOMO: highest occupied molecular orbital
ICG: indocyanine green
HRMS: high-resolution mass spectrometry
HT29: human colorectal adenocarcinoma cell line
LUMO: lowest unoccupied molecular orbital
LO2: human normal liver cell line
MB: methylene blue
MCF-7: human breast adenocarcinoma cell line
NQO1: NAD(P)H quinone oxidoreductase 1
NIR: near-infrared
NBS: N-Bromosuccinimide
SD: standard deviation
TNR: tumor-to-normal tissue ratio
TPA: two-photon absorption
TBAB: tetrabutylammonium bromide
THF: tetrahydrofuran
5-ALA: 5-Aminolevulinic acid
Ф_F_: fluorescence quantum yield
