# Supplementary material for: Novel pH-activatable NIR fluorogenic spray mediated near-instant and precise tumor margins identification in human cancer tissues for surgical resection
Source: Theranostics. 2023 Aug 15;13(13):4497–511. doi: 10.7150/thno.85651 (PMC10465228; doi:10.7150/thno.85651)
Supplement: Supplementary file 1 — Supplementary figures and tables. [file thnov13p4497s1.pdf]

Novel pH-activatable NIR fluorogenic spray mediated near-instant and precise tumor margins identification in human cancer tissues for surgical resection

Zhongyuan Xu [1,4,#], Jianqiang Qian [1,4,#], Hongmei Wu [1,4,#], Chi Meng [1], Qian Ding[1], Weizhi Tao [1,4], Chang-Chun Ling [2], Jun Chen [3], Peng Li [2], Yumin Yang [1,4,*], and Yong Ling [1,4,*]

[1]School of Pharmacy and Jiangsu Province Key Laboratory for Inflammation and Molecular Drug Target, Nantong University, 226001 Nantong, Jiangsu, PR China.

[2]Department of General Surgery, Affiliated Hospital of Nantong University, 226001 Nantong, Jiangsu, PR China.

[3]Department of Hepatobiliary surgery, Nantong Third People's Hospital and the Third Affiliated Hospital of Nantong University, 226001 Nantong, Jiangsu, PR China.

[4]Nantong Key Laboratory of Small Molecular Drug Innovation, Nantong University, 226001 Nantong, Jiangsu, PR China.

[#]These three authors contributed equally to this work.

*Corresponding author: E-mails: Lyyy111@ntu.edu.cn, yangym@ntu.edu.cn

Content

1. Experimental Section 3
1.1. Materials and instrument 3
1.2. Synthesis of **NBD** 3
1.3. pH-dependent optical spectra 5
1.4. Calculation of pKa 5
1.5. Fluorescence selectivity assessment 5
1.6. Calculation of fluorescence quantum yield (Φ_F) 5
2. Fluorescence quantum yield 6
3. Representative probes and NBD 6
4. Calculation of pKa 7
5. Protonation of β-carboline skeleton 7
6. Cell viability of normal cells 9
7. Intracellular lysosome colocalization 10
8. Cellular uptake 11
9. Optical properties of NBD 12
10. *Ex vivo* spray onto resected tumor and organs 13
11. Penetration depth of two-photon imaging 14
12. Metastasis-bearing model 14
13. Concentration screen for application to clinical specimens 15
14. *Ex vivo* spray onto clinical specimens 16
15. Large Stokes shift 18
16. Biosafety of NBD 19
17. Characterization of compounds 20
18. Reference 24

1. Experimental Section

1.1. Materials and instrument

Unless otherwise stated, all chemicals and reagents were commercially available and used without further purification. High Performance Liquid Chromatography (HPLC) was performed using Waters 1525 binary HPLC pump, Waters 2998 photodiode array detector, XBridge-C18 analytic column (5 μm, 4.6 mm × 150 mm). ^{1}H and ^{13}C NMR spectra were measured on a Bruker AV 400 MHz spectrometer. High-resolution mass spectra (HRMS) data were determined using an JMS-SX102A (FAB) or LC/MSD TOF. UV-visible absorption spectra were measured on a on a spectrometer (UV1800PC, Jinghua, China). Emission spectra were recorded on a fluorescence spectrophotometer (RF-6000, Shimadzu, Japan). Cell imaging was performed using Leica TCS SP8 LSM confocal microscope equipped with a 63× oil plan apochromatic objective. The *in vivo* and tissue imaging were performed by IVIS Lumina XR Series III optical imaging system. Compounds **4** were prepared according to previously reported methods [S1-2].

1.2. Synthesis of **NBD**

*Synthesis of compound **5***

To a solution of compound **4** (4.5 g, 17.72 mmol) in 50 mL THF was added NBS (3.78 g, 21.24 mmol) and TBAB (6.85g, 21.24 mmol). The reaction was stirred at 45 °C for 1 h. The precipitate was collected through filtration to afford compound **5** as yellow solid (5.37 g, 91%). ^{1}H NMR (400 MHz, $CDCl_3$) δ 8.64 (s, 1H, ArH), 8.31 – 8.13 (m, 1H, ArH), 7.75 – 7.61 (m, 1H, ArH), 7.34 (d, *J* = 8.8 Hz, 1H, ArH), 4.14 (s, 3H, CH_3), 4.05 (s, 3H, CH_3), 3.14 (s, 3H, CH_3).

*Synthesis of compound **6***

A solution of compound **5** (5.30 g, 15.9 mmol) in 50 mL anhydrous THF was cooled to -5 °C for 20 min before $LiAlH_4$ (741 mg, 19.5 mmol) was slowly added, then the reaction was stirred at 27 °C for 30 min. The reaction was restored to 27 °C and then stirred at 27 °C for another 3 h. Methanol was added until no bubbles could be observed and then 1 mL H_2O was added. Then the mixture was filtered and washed with methanol. The collecting filtrate was concentrated under reduced pressure to afford compound **6** as yellow solid (3.3 g, 68%).

*Synthesis of compound **7***

To a solution of compound **6** (3.3 g, 10.82 mmol) in 30 mL DCM was added DMP (10.8

g, 55.67 mmol). About 2 h later, 50 mL sodium bicarbonate and sodium thiosulfate were slowly added to the mixture which was then extracted with DCM (100 mL × 3). The combined organic layer was dried over sodium sulfate and concentrated under reduced pressure to afford compound **7** as yellow solid (2.2 g, 67%).

Synthesis of compound ***8***

To a solution of compound **7** (2.2 g, 7.25 mmol), diphenylamine (2.45 g, 14.48 mmol), $Pd(dba)_2$ (0.8338g, 1.45 mmol) and sodium tert-butoxide (1.4 g, 14.57 mmol) in 0.1 mL tri-tert-butylphosphine was added 4 mL toluene. The reaction was stirred at 110 °C for 12 h. The reaction solution was quenched with NH_4Cl and extracted with ethyl acetate for three times. The organic layer was evaporated under reduced pressure and recrystallized to afford compound **8** as yellow solid (2.15 g, 76%). ^{1}H NMR (400 MHz, $CDCl_3$) δ 10.03 (s, 1H, CHO), 8.32 (s, 1H, ArH), 7.73 (d, *J* = 2.3 Hz, 1H, ArH), 7.39 – 7.29 (m, 4H, 4ArH), 7.00 – 6.97 (m, 4H, 4ArH), 6.93 – 6.89 (m, 4H, ArH), 4.11 (s, 3H, CH_3), 3.07 (s, 3H, CH_3). ^{13}C NMR (101 MHz, DMSO-*d6*) δ 168.5, 148.5, 143.0, 140.1, 138.0, 129.7, 127.7, 126.6, 123.8, 123.3, 122.6, 122.1, 122.0, 119.6, 111.3, 109.4, 29.0, 14.3. HRMS: (ESI, *m/z*) Calcd for $C_{26}H_{21}N_3O$ $[M]^+$: 392.1763, found 392.1766.

Synthesis of compound ***10***

To a solution of compound **9** (2 g, 9.56 mmol) in 20 mL methanol was added CH_3I (2.71g, 19.12 mmol). The reaction was stirred at 80 °C for 8 h. The mixture was evaporated under reduced pressure and recrystallized with methanol to afford compound **10** as light gray solid (3.3 g, 98%).

Synthesis of ***NBD***

To a solution of compound **8** (2 g, 5.11 mmol) and compound **10** (1.79 g, 5.11 mmol) in ethanol was added two drops of piperidine. The reaction was stirred at 80 °C for 3 h. The mixture was concentrated under reduced pressure and recrystallized with methanol. The precipitate was filtered to afford **NBD** as atroceruleous solid (3.2 g, 87%). ^{1}H NMR (400 MHz, $CDCl_3$) δ 8.50 – 8.42 (m, 2H, CH=C, ArH), 8.20 (d, *J* = 9.2 Hz, 1H, ArH), 8.12 – 8.09 (m, 2H, ArH, CH=C), 8.07 – 8.04 (m, 1H, ArH), 7.98 (d, *J* = 1.8 Hz, 1H, ArH), 7.92 (d, *J* = 8.9 Hz, 1H, ArH), 7.75 – 7.71 (m, 1H, ArH), 7.67 – 7.62 (m, 1H, ArH), 7.51 – 7.45 (m, 2H, 2ArH), 7.32 – 7.27 (m, 4H, 4ArH), 7.13 – 7.09 (m, 4H, 4ArH), 7.04 – 7.00 (d, *J* = 7.7 Hz, 2H, 2ArH), 4.52 (s, 3H, CH_3), 4.24 (s, 3H, CH_3), 3.17 (s, 3H, CH_3), 2.12 (s, 6H, $2CH_3$).^{13}C NMR (101 MHz, DMSO-*d6*) δ 182.4, 154.0, 148.5, 143.5, 141.1, 140.0, 139.7, 137.9, 133.4, 131.2, 131.0, 130.5, 130.2, 129.9, 129.7, 128.8, 127.3, 127.2, 127.0, 126.7, 126.3, 124.7, 124.7, 123.8, 123.6, 122.9, 122.6, 122.5, 121.6, 113.6, 113.0, 112.8, 110.9, 110.7, 109.8, 53.9, 38.2, 35.2, 26.0, 14.3. HRMS: (ESI, *m/z*) Calcd for $C_{42}H_{37}N_4^+$ $[M]^+$: 597.3013, found 597.3016.

1.3. pH-dependent optical spectra

The solution of **NBD** (10 μM) in deionized water (containing 25% v/v methanol) was first prepared. All absorption and fluorescence spectra were determined in a 1 cm standard quartz cell (3 mL volume) with pH ranging from 4.01 to 7.38 or 3.56 to 7.40. Emission spectra were excited at 582 nm, and recorded from 650 to 850 nm.

1.4. Calculation of pKa.

pKa was calculated following the equation:

$$pKa = pH - \log_{10}[(I_{max}-I)/(I - I_{min})]$$

where I is the fluorescence intensity of **NBD** at 742 nm, I_{max} and I_{min} are the maximum and minimum values of fluorescence intensity.

1.5. Fluorescence selectivity assessment

The solutions of **NBD** (10 μM) in deionized water (containing 25% v/v methanol) at 37 °C was respectively mixed with Na^+, K^+, Cu^{2+}, Fe^{2+}, Zn^{2+}, H_2O_2, OCl^-, GSH, VcNa, NQO1 and NTR (1 mM) for 0.5 h or adjusted to pH = 4.0. The fluorescence intensity at 742 nm was collected with excitation wavelength at 582 nm.

1.6. Calculation of fluorescence quantum yield (Φ_F)

The fluorescence quantum yield of **NBD** at pH= 7.4 and pH = 4.0 was determined in ethanol with Rhodamine B (Φ_F=0.49, in ethanol) as a reference [S3]. Φ_F was determined according to the following equation in the literature [S4]:

$$\Phi_{F(X)}=\Phi_{F(S)}\,(A_S F_X/A_X F_S)\,(n_X/n_S)^2$$

where Φ_F is fluorescence quantum yield. F is the integrated area under fluorescence spectrum at the same excitation wavelength. A is the absorbance at the excitation wavelength. S and X denote reference and testing sample. n is the refractive index of a specific solvent (n_X/n_S=1 in this work).

2. Fluorescence quantum yield

Table S1. Fluorescence quantum yield (Φ_F) of NBD at different pH values.

**Compound**	**Area**	**Φ_F**
Rhodamine B	20655	0.49
**NBD** at pH = 7.4	1988	0.047
**NBD** at pH = 4.0	11343	0.26

3. Representative probes and NBD

Table S2. Representative probes employed to distinguish tumor tissue and normal tissues via spraying manner.

**Probe**	Waiting time	Reaction mechanism	Imaging application models	Reference
**APN-ACLP**	~10 min	Aminopeptidase N-activatable chemiluminescent probe	Tumor-bearing mice	S5
**PC-EcN-NTR**	~30 min	Nitroreductase-activatable fluorescent Probe	Tumor-bearing mice	S6
**YH-APN**	~30 min	Aminopeptidase N-activatable fluorescent Probe	Tumor-bearing mice	S7
**AzaB5**	~30s	Acidic TME-activatable fluorescent probe	Tumor-bearing mice	S8
**CypH-11**	~7 min	Acidic TME-activatable fluorescent probe	Tumor-bearing mice	S9
**NIR-SN-GGT**	~30min	γ -glutamyltranspeptidase-activatable fluorescent probe	Tumor-bearing mice	S10
**EP-HMRG**	~10 min	dipeptidylpeptidase IV-activatable fluorescent probe	Clinical tissues	S11
**Z-Phe-Arg-HMRG**	~7 min	cathepsin-activatable fluorescent probe	Tumor-bearing mice	S12
**gGlu-HMRG**	>10 min	γ -glutamyltranspeptidase-activatable fluorescent probe	Tumor-bearing mice	S13
**P-CyPt**	~30 min	alkaline phosphatase-activatable fluorescent probe	Tumor-bearing mice	S14
**NBD** (this work)	~3 min	Acidic TME-activatable fluorescent probe	Clinical tissues	/

4. Calculation of pKa.

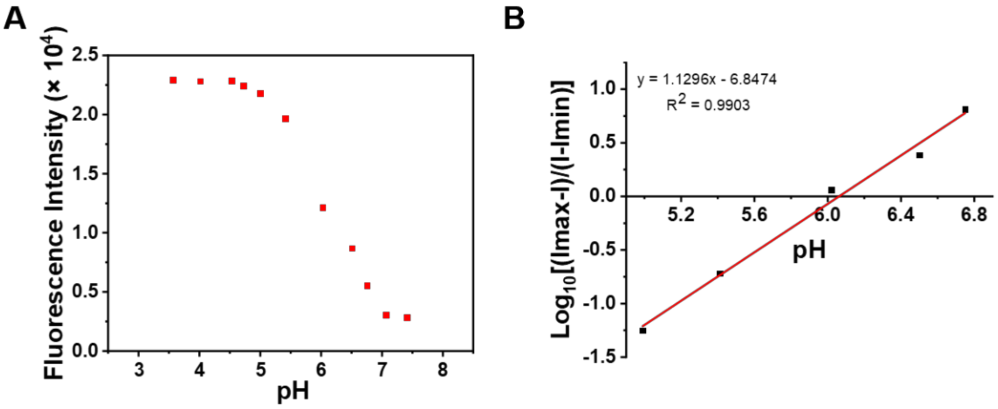

Figure S1. Calculation of pKa in deionized water (25% v/v methanol). (A) Fluorescence intensity−pH titration profiles of **NBD** at wavelength of 742 nm. (B) Calculation of pKa.

5. Protonation of β-carboline skeleton

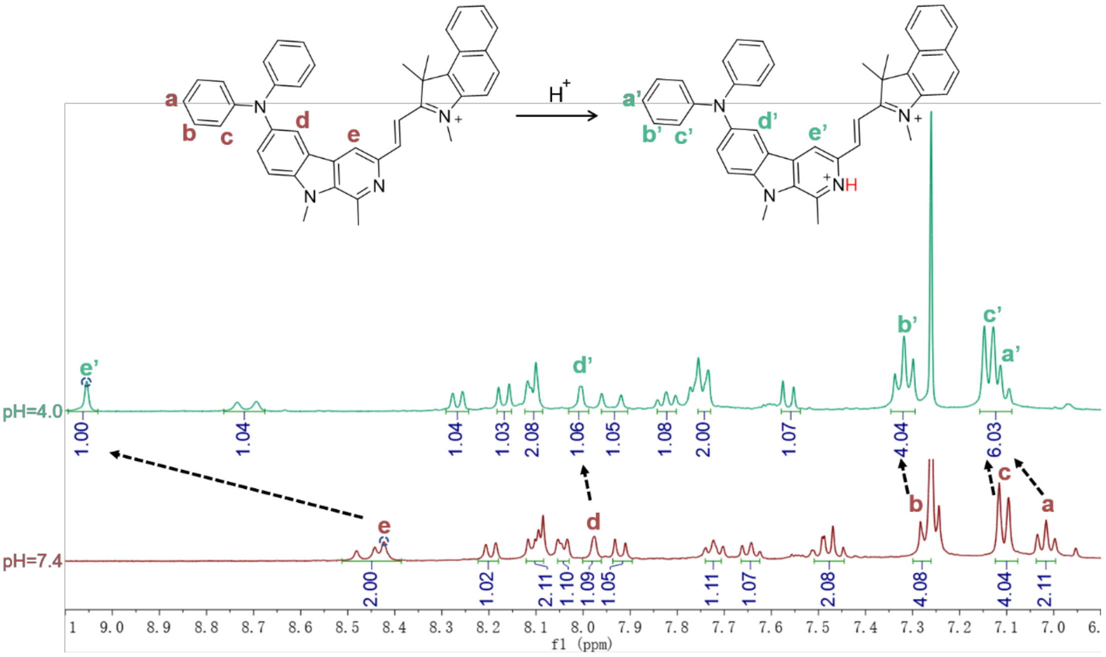

Figure S2. ^{1}H NMR spectra (400 MHz) of **NBD** (20 mg/mL) in the region of 9.10−6.90 ppm upon addition of deuterated TFA to adjust pH=4.0 (the upper spectrum) and pH=7.4 (the lower spectrum) in $CDCl_3$.

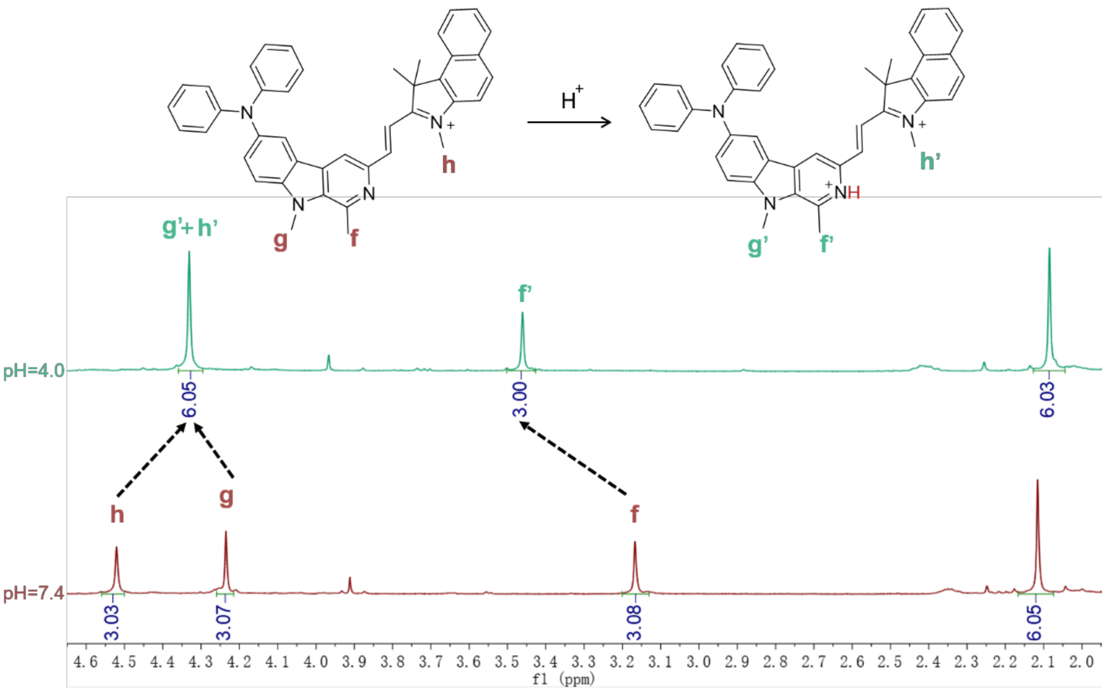

Figure S3. ^{1}H NMR spectra (400 MHz) of **NBD** (20 mg/mL) in the region of 4.65−1.95 ppm upon addition of deuterated TFA to adjust pH=4.0 (the upper spectrum) and pH=7.4 (the lower spectrum) in $CDCl_3$.

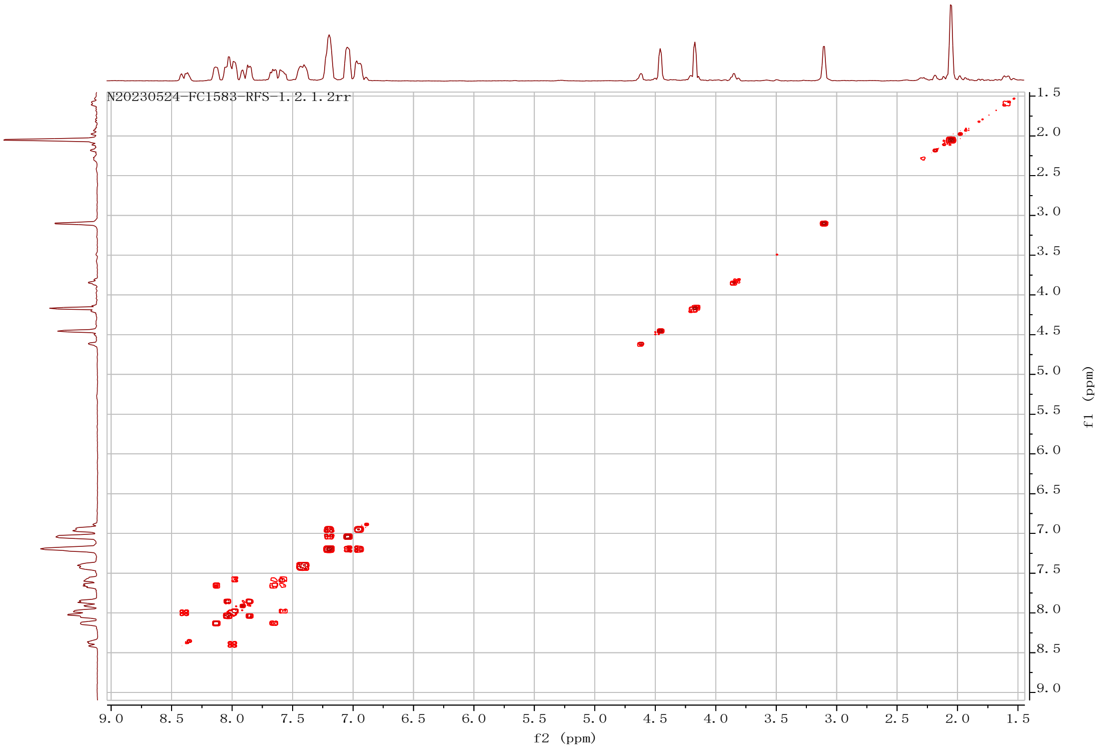

Figure S4. ^{1}H-^{1}H COSY NMR spectrum of **NBD** at pH=7.4 in $CDCl_3$.

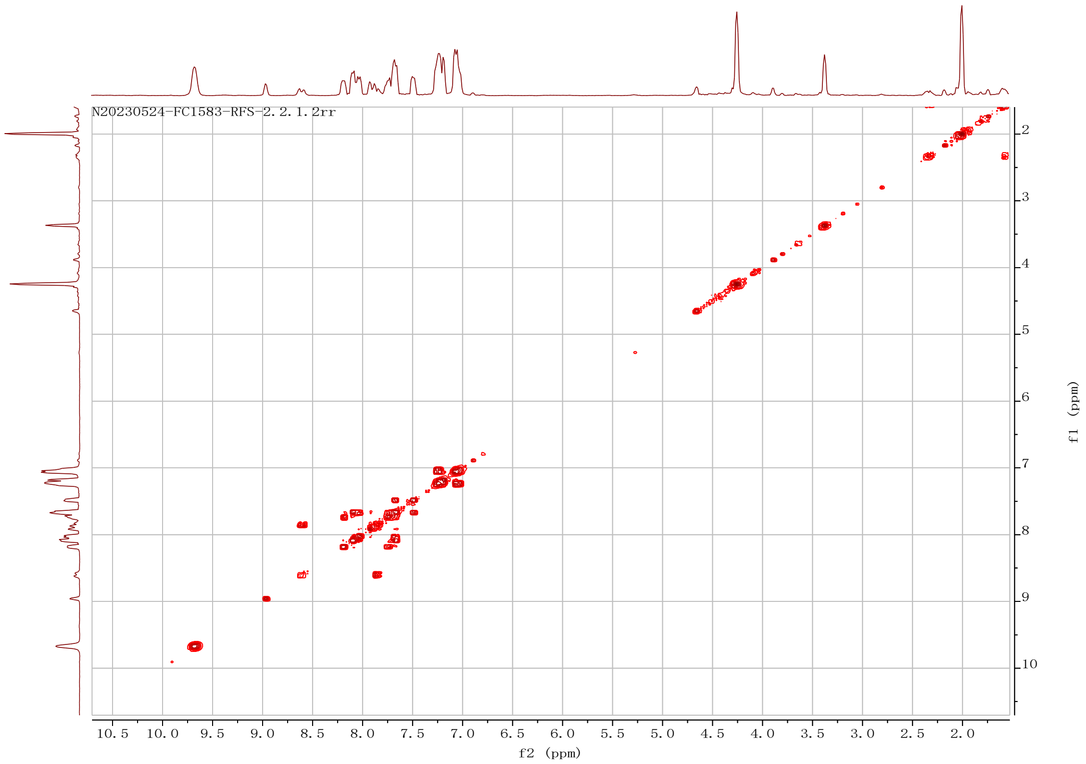

Figure S5. ^{1}H-^{1}H COSY NMR spectrum of **NBD** at pH=4.0 in $CDCl_3$.

6. Cell viability of normal cells

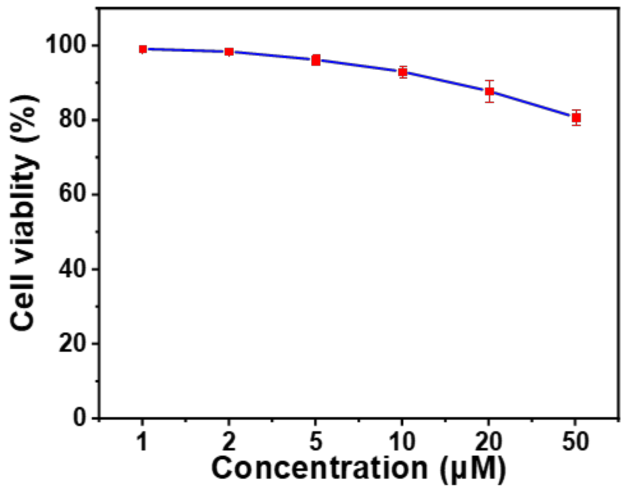

Figure S6. Cell viability of normal cells (LO2) treated with different concentrations of **NBD**. (mean ± SD, n = 3)

7. Intracellular lysosome colocalization

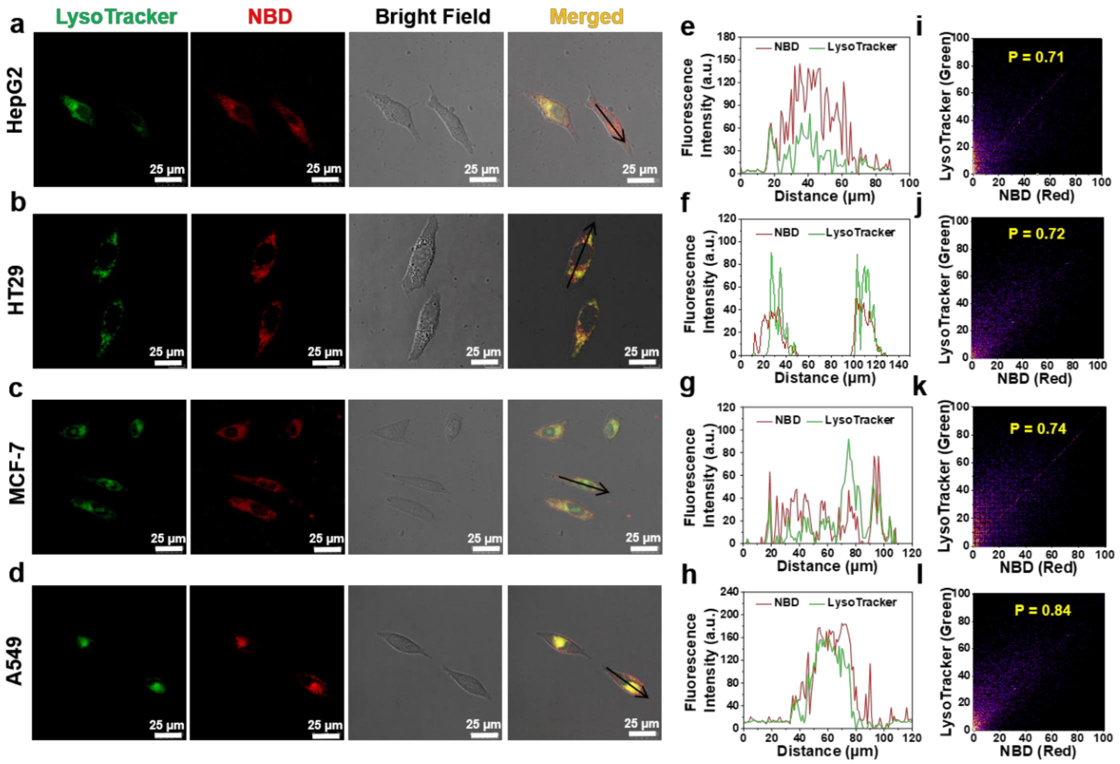

Figure S7. Intracellular lysosome colocalization. (A-D) HT29 (A), HT29 (B), MCF-7 (C), A549 (D) cells were pretreated with **NBD** for 1 h and then incubated with Lysotracker Green for 30 min. (E-H) The intensity profiles along with the black arrow of cancer cells from (A)-(D). (I-L) Plots representing the intensity correlation of Lysotracker Green and **NBD**. **NBD** was excited at 600 nm. LysoTracker Green were excited at 488 nm. Scale bar = 25 μm

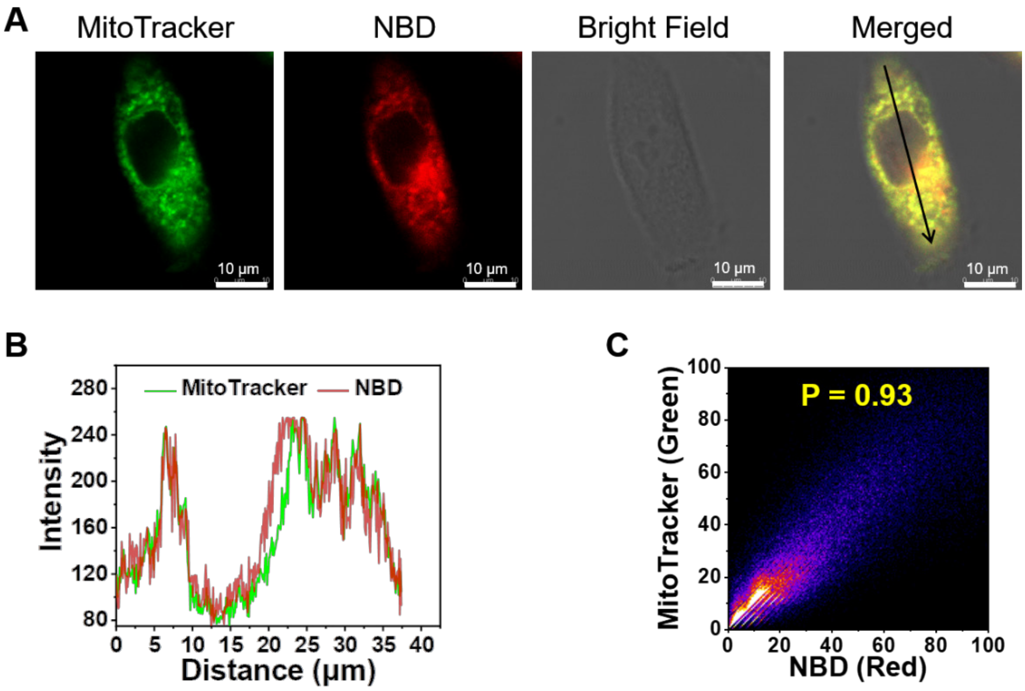

Figure S8. **Mitochondrion colocalization of NBD in HepG2 cells**. (A) Confocal images of HepG2 stained with **NBD**/MitoTracker using Leica TCS SP8 LSM confocal microscope equipped with a 63× oil plan apochromatic objective. (B) The intensity profiles along with the black arrow of the cells. (C) Intensity correlation plot of **NBD** and Mitotracker Green.

8. Cellular uptake

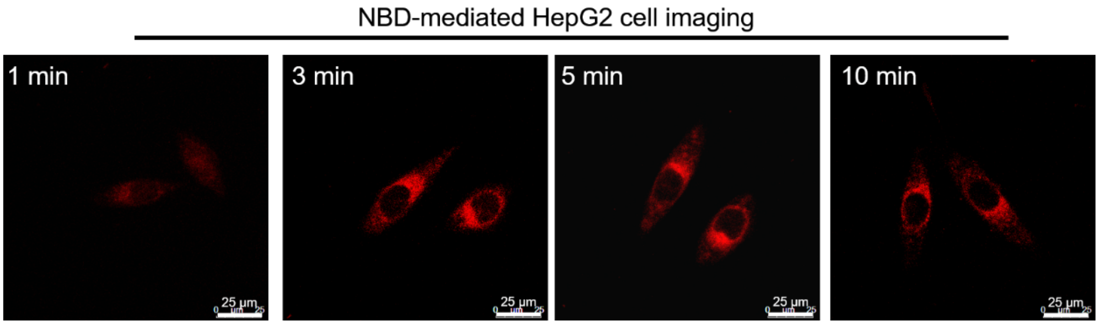

Figure S9. Time-dependent fluorescence images of HepG2 after incubation with **NBD** (5 μM) for 1, 3, 5, 10 min.

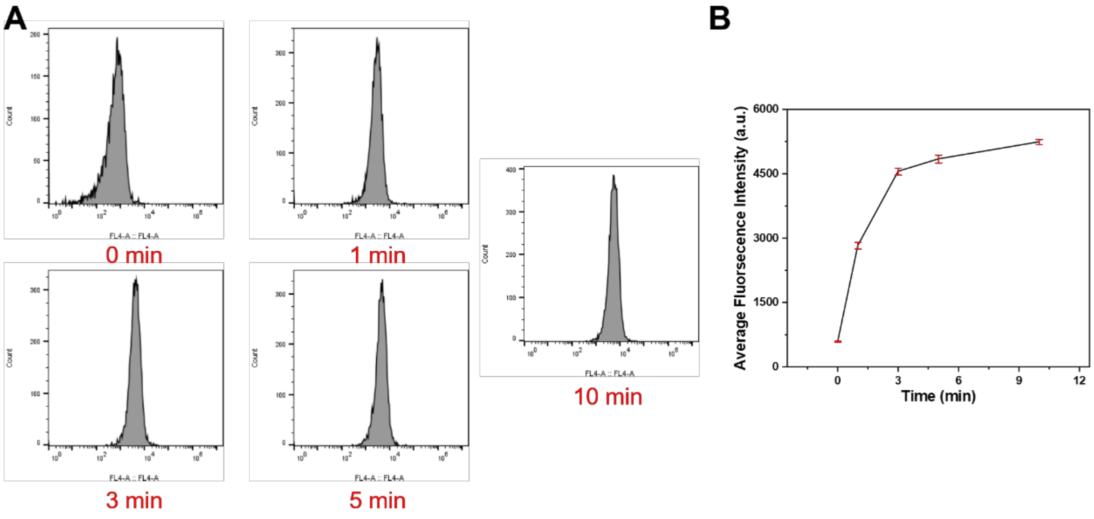

Figure S10. **NBD uptake by HepG2 cells.** (A) Time-dependent fluorescence enhancement of HepG2 cells after incubation with **NBD** (5 μM) for 1, 3, 5, 10 min via flow cytometry analysis. (B) Average fluorescence intensity at different time.

9. Optical properties of NBD

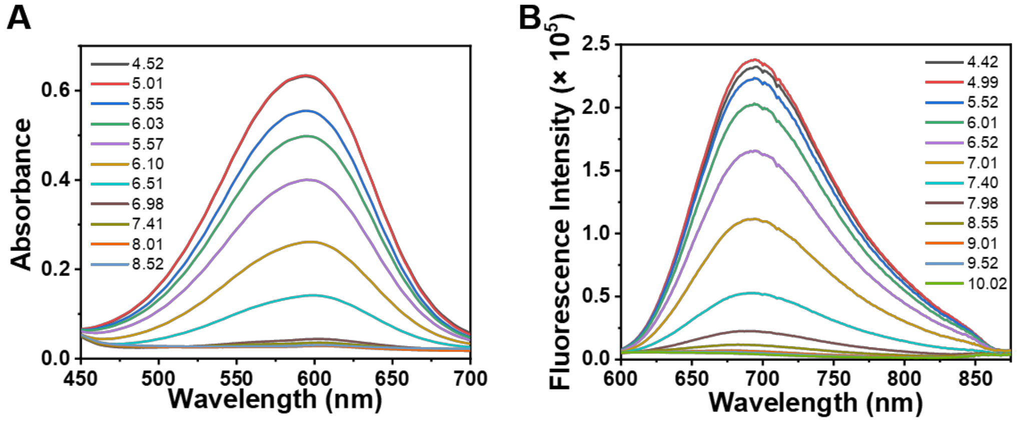

Figure S11. **Optical properties of NBD in saline (10 μM, 5% DMSO, and 10% Tween 80).** (A,B) Absorption spectra (A) and fluorescence spectra (B) of **NBD** at different pH.

10. *Ex vivo* spray onto resected tumor and organs

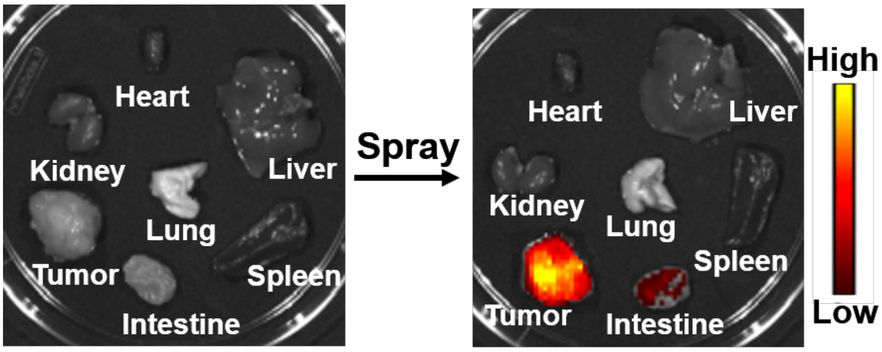

Figure S12. Representative fluorescence images of the excised tumors and normal organs in xenograft tumor nude mice after spraying **NBD**.

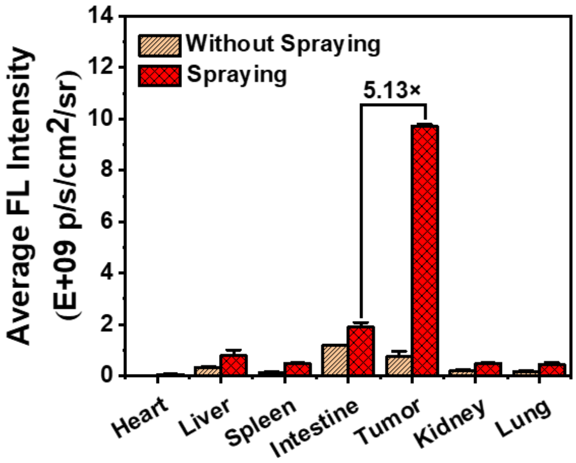

Figure S13. The relative average fluorescence intensity of tumors and organs resected from tumor xenograft nude mice before and after spraying. (mean ± SD, n = 3).

11. Penetration depth of two-photon imaging

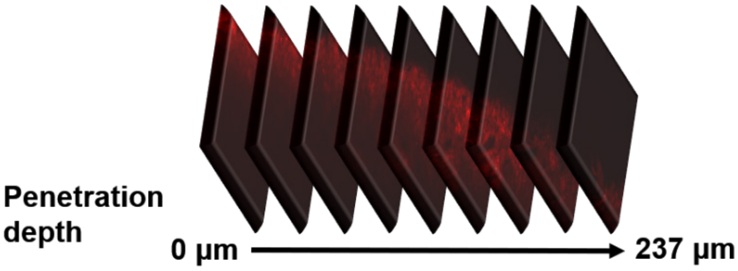

Figure S14. Sectional TPM images of colon cancer tissues labeled with **NBD,** with a depth ranging from 0 μm to 237 μm. (λ_{ex} = 1040 nm for two-photo excitation)

12. Metastasis-bearing model

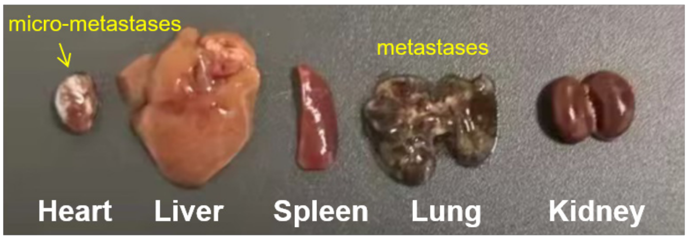

Figure S15. Representative photograph of main organs from metastasis-bearing mice.

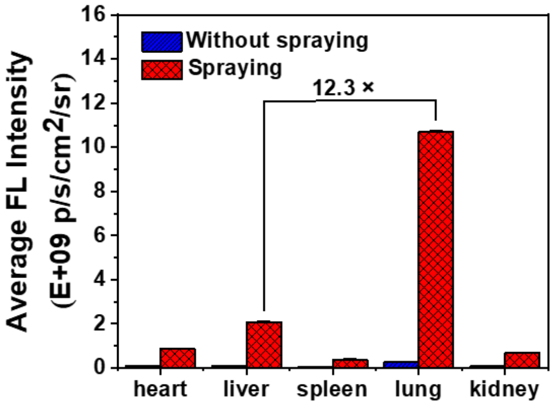

Figure S16. The relative average fluorescence intensity of the main organs resected from 4T1 metastasis-bearing models before and after spraying (mean ± SD, n = 3).

13. Concentration screen for application to clinical specimens

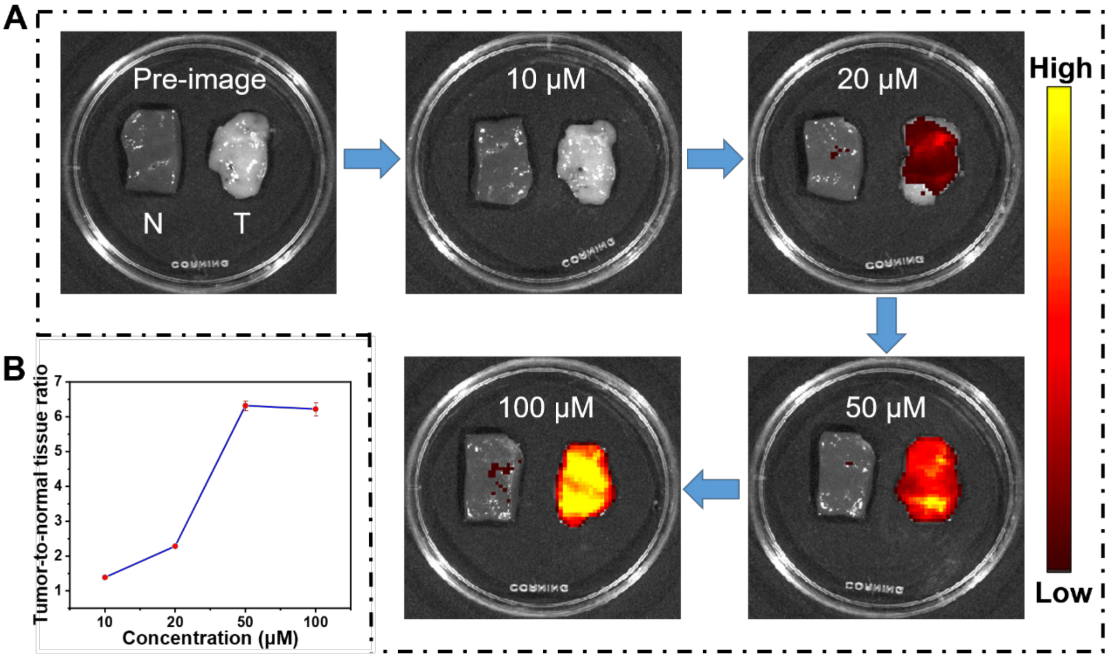

Figure S17. NBD concentration screen. (A) Representative fluorescence images of clinical tissues after spraying of **NBD** at various concentrations. (B) TNR of fluorescence according to (A). T: tumor tissue, N: normal tissue.

14. *Ex vivo* spray onto clinical specimens

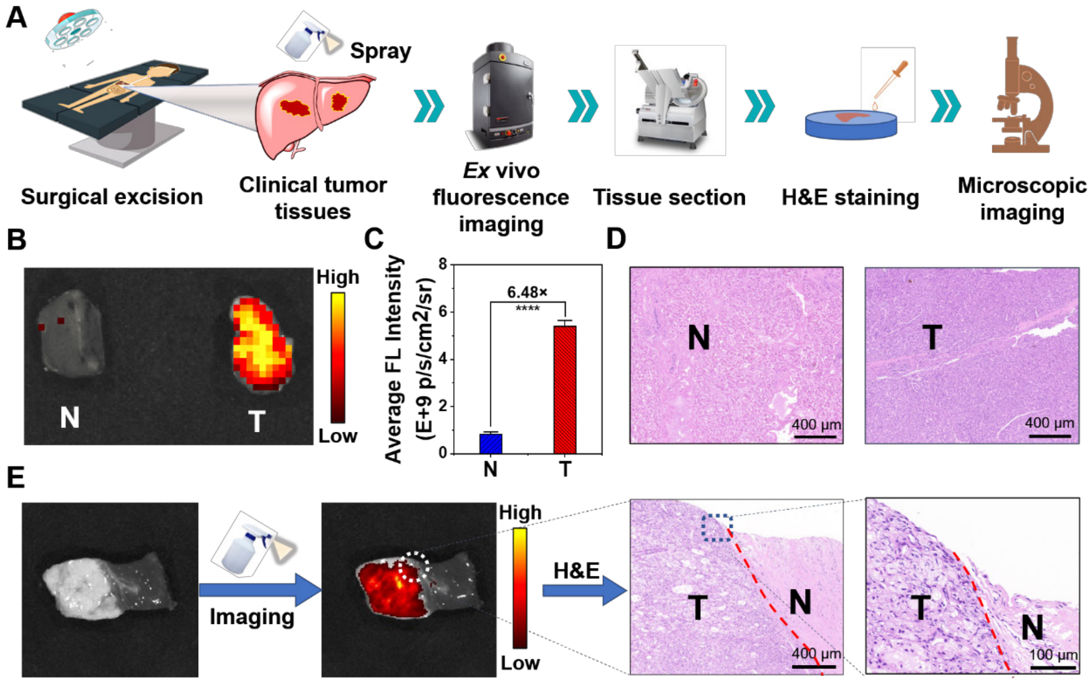

Figure S18. *Ex vivo* spray for clinical liver cancer identification. (A) Schematic illustration displaying liver cancer detection method in human specimens. (B) Fluorescence analysis of clinical liver tissue and normal tissue. (C) Quantification of the fluorescence intensity according to (B). (D) H&E analysis of normal tissue and tumor tissue. (E) Representative fluorescence image of *ex vivo* clinical liver tissues after spraying with **NBD** (50 μM) and H&E analysis of the fluorescence-indicated cancer margins. (****, $P<0.0001$)

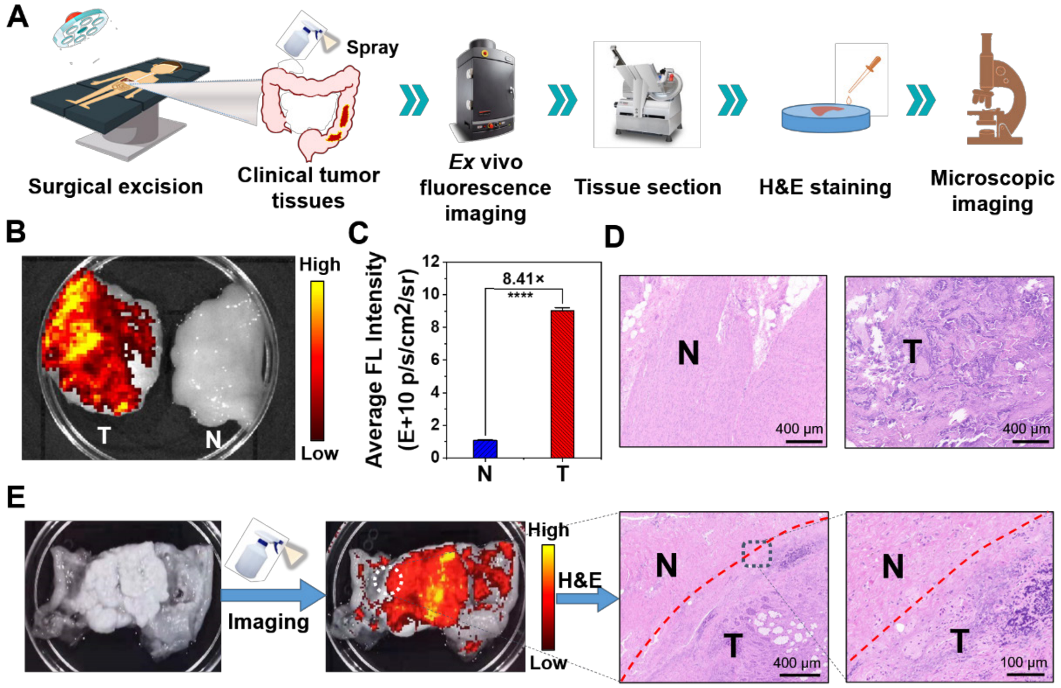

Figure S19. ***Ex vivo*** **spray for clinical colon cancer identification.** (A) Schematic illustration displaying colon cancer detection method in human specimens. (B) Fluorescence analysis of clinical colon tissue and normal tissue. (C) Quantification of the fluorescence intensity according to (B). (D) H&E analysis of normal tissue and tumor tissue. (E) Representative fluorescence image of *ex vivo* clinical liver tissues after spraying with **NBD** (50 μM) and H&E analysis of the fluorescence-indicated cancer margins. (****, P<0.0001)

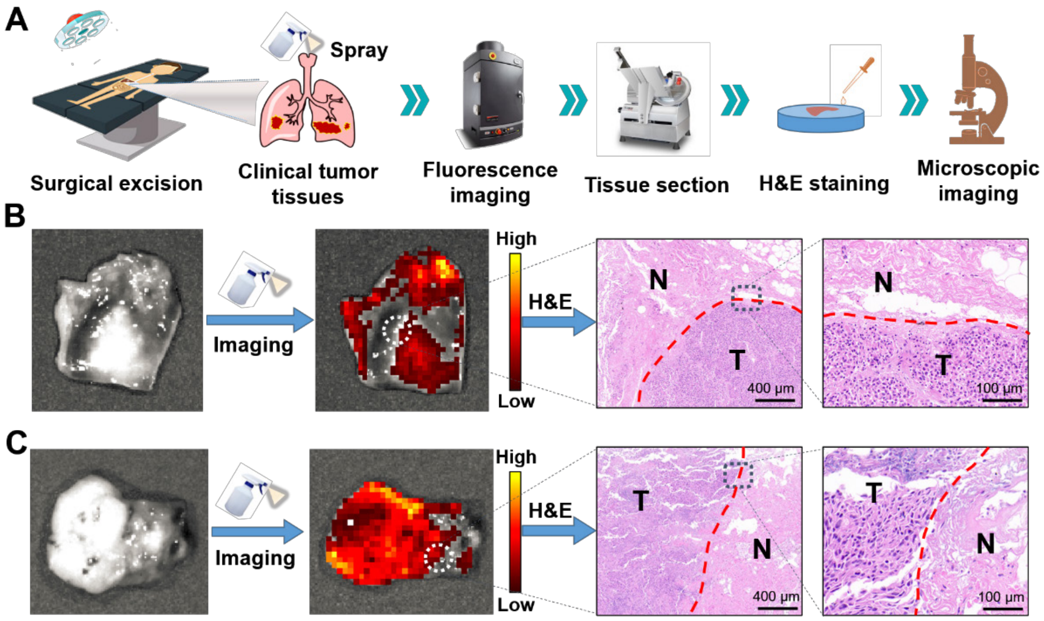

Figure S20. ***Ex vivo*** **spray for clinical lung cancer identification.** (A) Schematic illustration of lung cancer detection method in human specimens. (B–C) Representative fluorescence images of *ex vivo* clinical lung tissues after spraying with **NBD** (50 μM) and H&E analysis of the fluorescence-indicated cancer margins.

15. Large Stokes shift

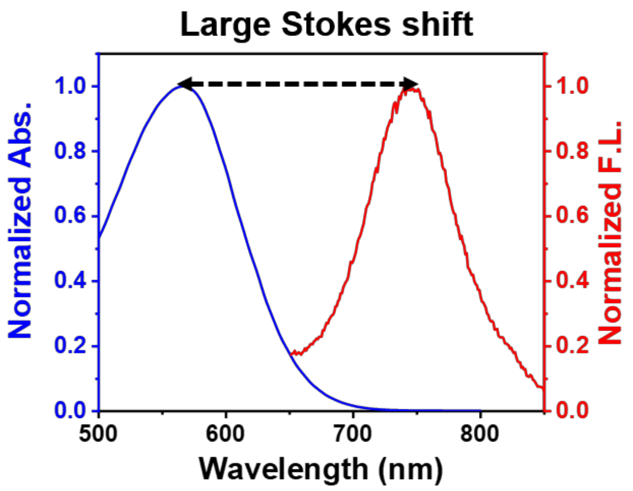

Figure S21. Absorption and fluorescence emission spectra of **NBD** at pH = 4.0. Large Stokes shift alleviated crosstalk between absorption and emission spectra, minimized self-quenching caused by reabsorption, and enhanced signal readout in tumor site.

16. Biosafety of NBD

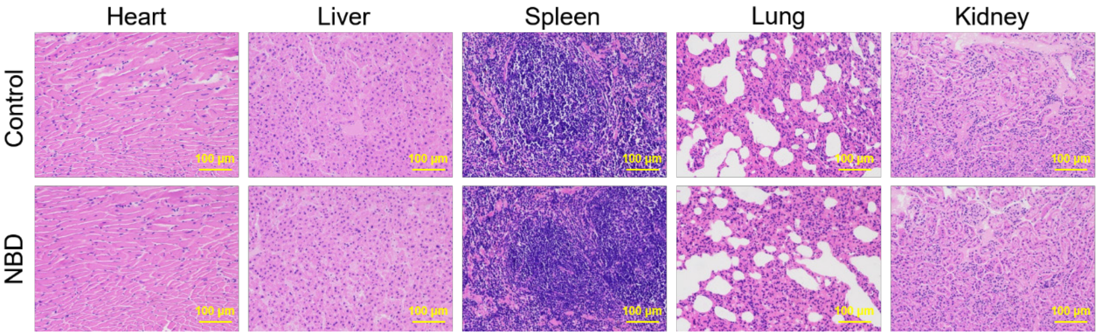

Figure S22. Histological images of the major organs (heart, liver, spleen, lungs, and kidneys) of female nude mice at 24 h after intravenous injection of saline and **NBD** with a dose of 1.1 mg/kg.

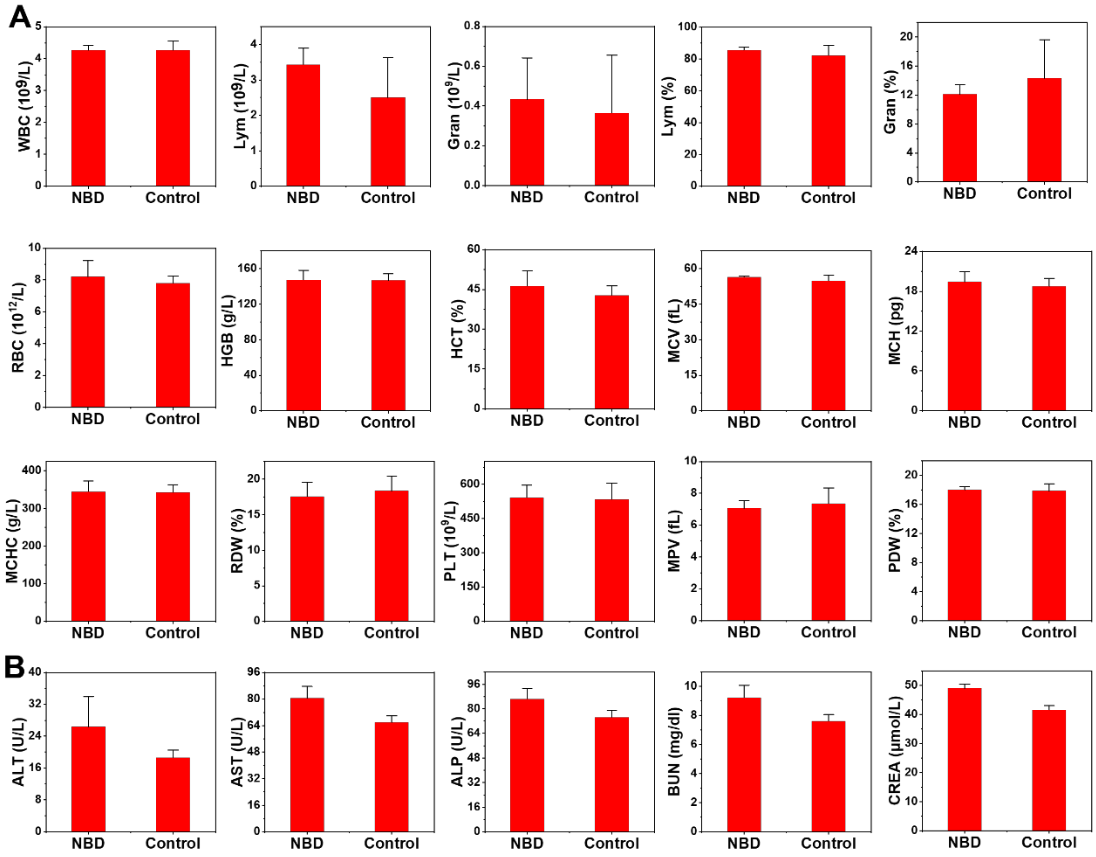

Figure S23. ***In vivo* safety evaluation of NBD**. (A) Assessment of blood routine examination results including white blood cells (WBC), lymphocyte cells (Lym), granulocyte cells (Gran), red blood cell (RBC), hemoglobin (HGB), red blood cell specific volume (HCT), mean corpuscular volume (MCV), mean corpuscular hemoglobin (MCH), mean corpuscular hemoglobin concentrations (MCHC), red cell volume distribution width (RDW), platelet (PLT), mean platelet volume (MPV), platelet distribution width (PDW). (B) Assessment of blood biochemistry indexes

including alanine aminotransferase (ALT), aspartate aminotransferase (AST), alkaline phosphatase (ALP), blood urea nitrogen (BUN) and creatinine (CREA).

17. Characterization of compounds

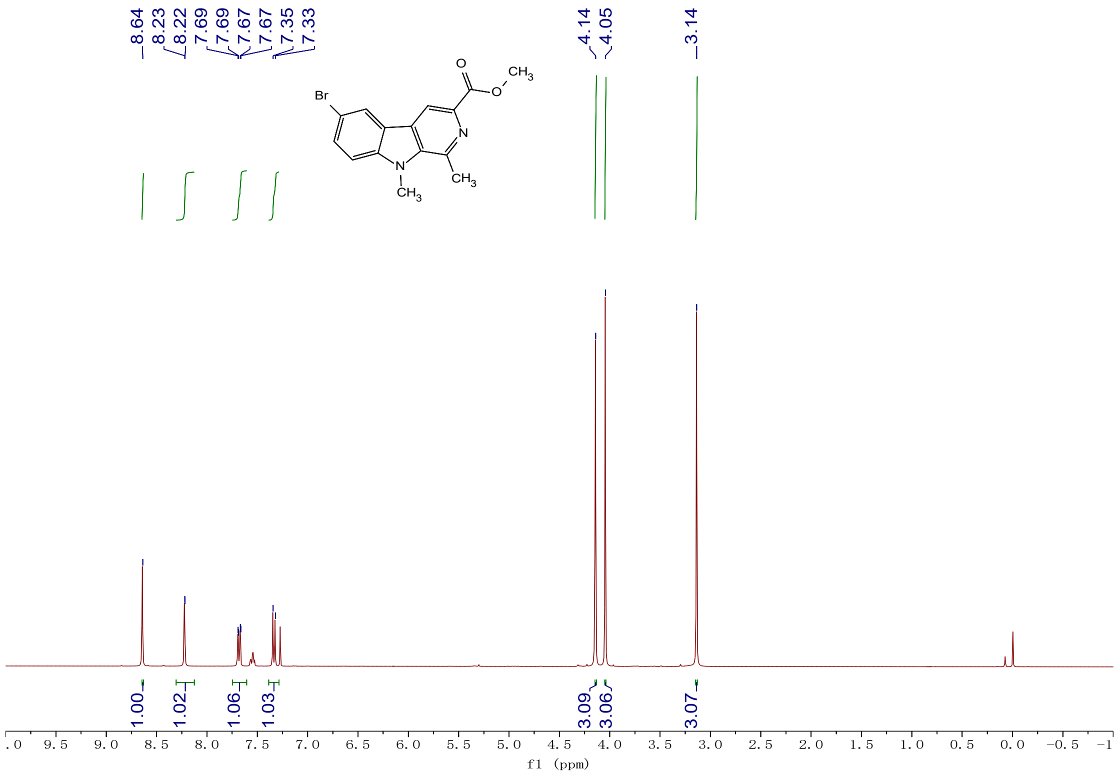

Figure S24. ^{1}H NMR spectrum of compound **5** in $CDCl_3$.

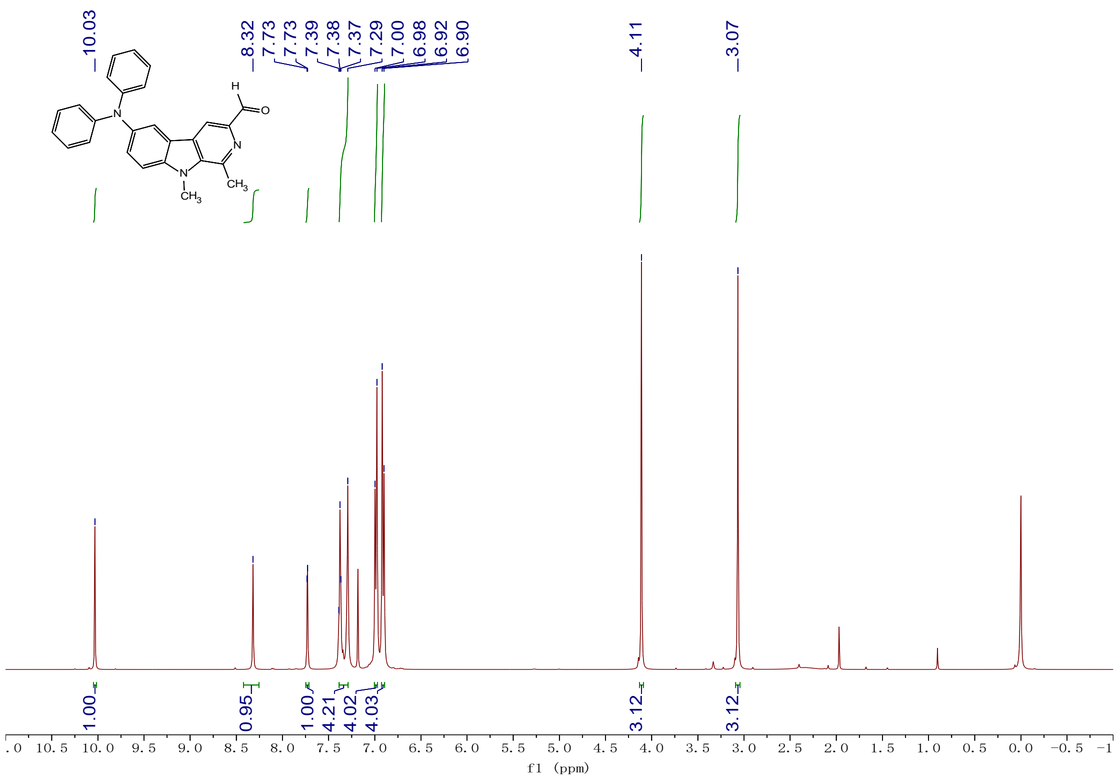

Figure S25. ^{1}H NMR spectrum of compound **8** in $CDCl_3$.

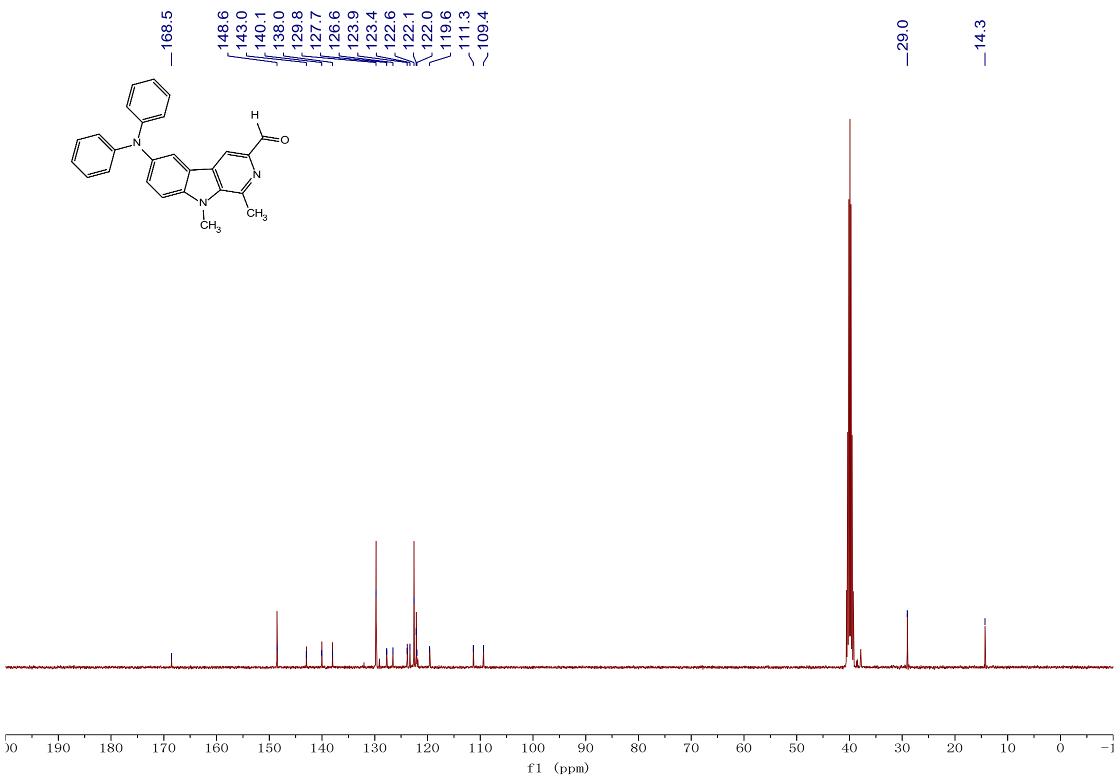

Figure S26. ^{13}C NMR spectrum of compound **8** in DMSO-*d6*.

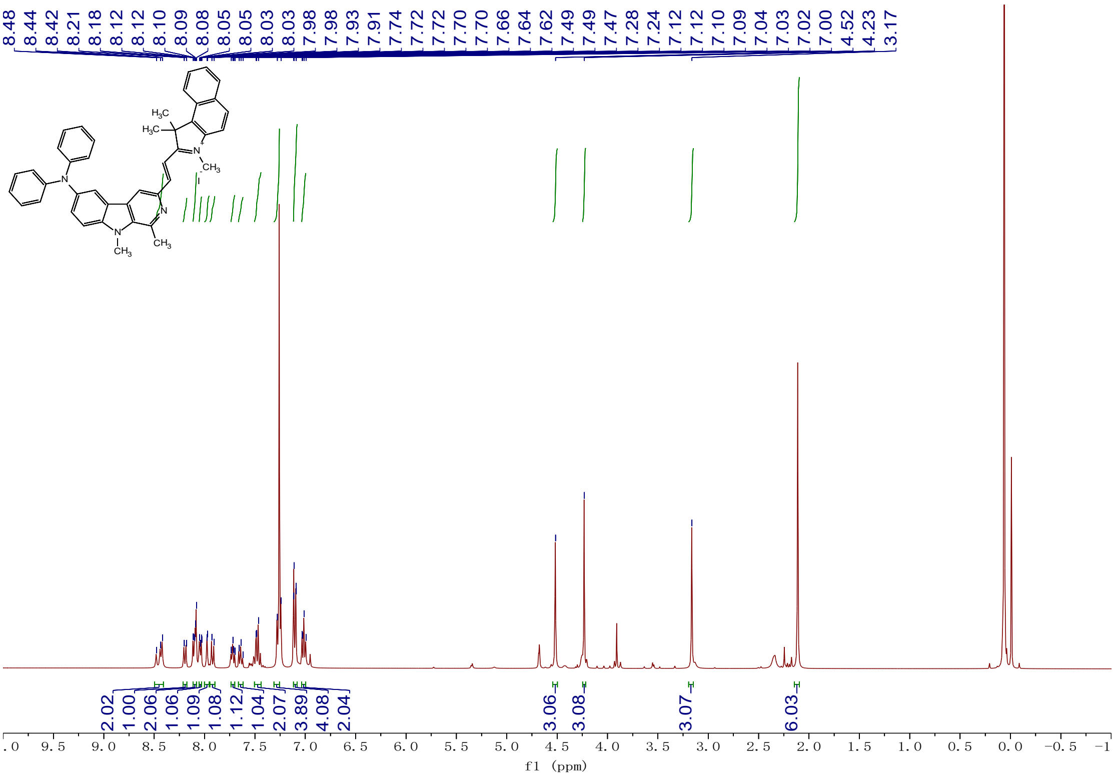

Figure S27. ^{1}H NMR spectrum of **NBD** in $CDCl_3$.

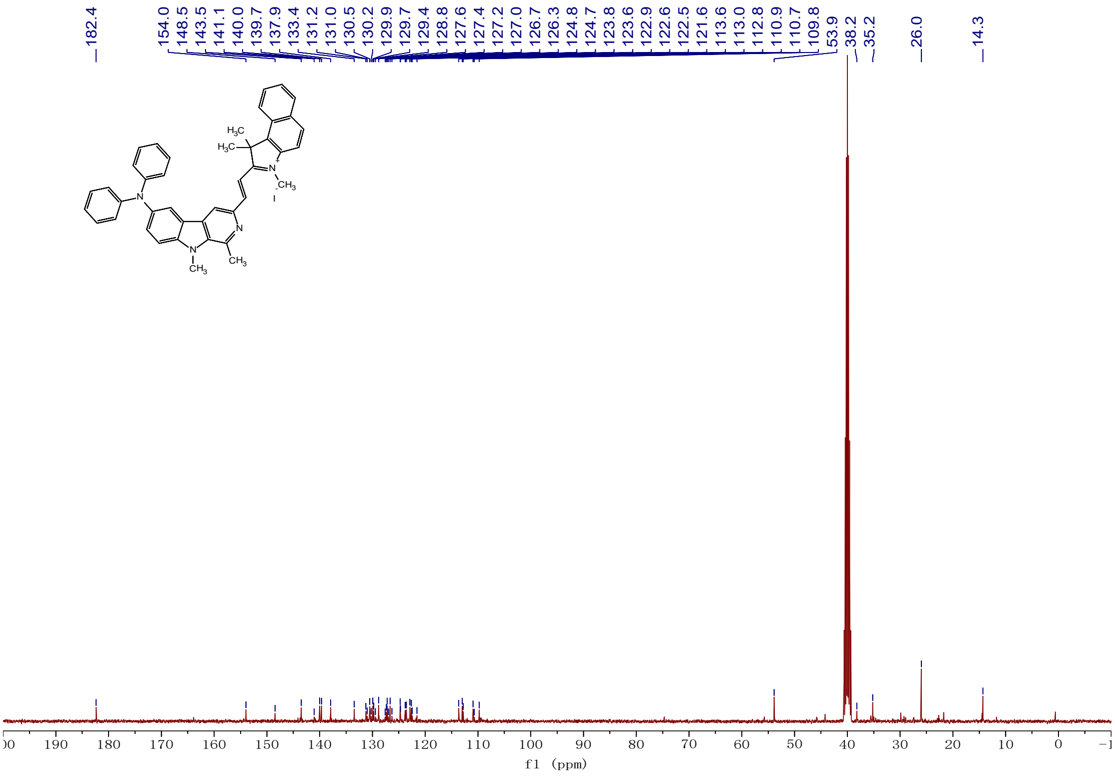

Figure S28. ^{13}C NMR spectrum of **NBD** in DMSO-*d6*.

Elemental Composition Report

Page 1

Single Mass Analysis
Tolerance = 10.0 PPM / DBE: min = -1.5, max = 50.0
Element prediction: Off
Number of isotope peaks used for i-FIT = 3

Monoisotopic Mass, Even Electron Ions
334 formula(e) evaluated with 1 results within limits (all results (up to 1000) for each mass)
Elements Used:
C: 26-26 H: 0-200 N: 0-20 O: 0-21
15
0611-1-6 112 (0.669)

1: TOF MS ES+
9.73e+002

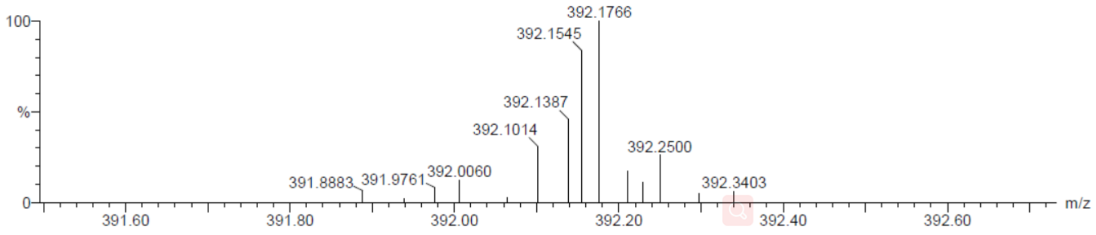

Minimum:				-1.5				
Maximum:		5.0	10.0	50.0				
Mass	Calc. Mass	mDa	PPM	DBE	i-FIT	Norm	Conf(%)	Formula
392.1766	392.1763	0.3	0.8	17.5	70.5	n/a	n/a	C26 H22 N3 O

Figure S29. HRMS spectrum of compound **8**.

Elemental Composition Report

Page 1

Single Mass Analysis
Tolerance = 5.0 PPM / DBE: min = -1.5, max = 50.0
Element prediction: Off
Number of isotope peaks used for i-FIT = 3

Monoisotopic Mass, Even Electron Ions
145312 formula(e) evaluated with 1 results within limits (up to 50 closest results for each mass)
Elements Used:
C: 42-42 H: 37-37 B: 0-1 N: 4-4 O: 0-100 Cu: 0-5 Se: 0-1 Br: 0-8 I: 0-1

A
1130-1-WC-23 127 (0.723)

1: TOF MS ES+
9.40e+001

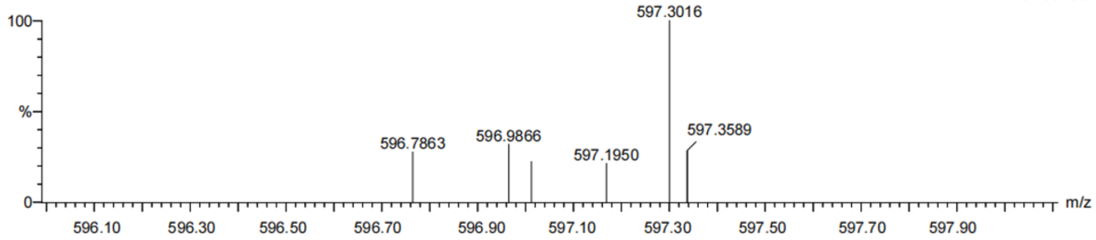

Minimum				-1.5				
Maximum:		5.0	5.0	50.0				
Mass	Calc Mass	mDa	PPM	DBE	i-FIT	Norm	Conf(%)	Formula
597.3016	597.3016	-0.4	-0.5	26.5	49.6	n/a	n/a	C42 H37 N4

Figure S30. HRMS spectrum of **NBD**.

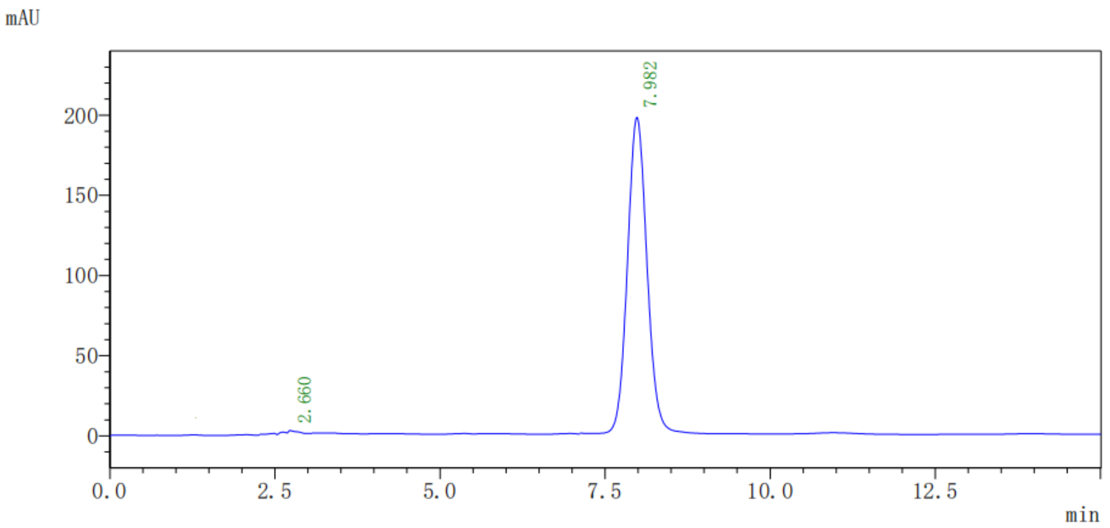

Figure S31. HPLC analysis of **NBD. NBD**: Ret time = 7.982 min, purity 98%. HPLC analysis methods: column: Shimadzu C18 (150 mm×4.6 mm×5 μm); mobile phase: methanol (0.1% formic acid): water = 80:20; wavelength: 254 nm; flow rate: 1 mL/min.

18. Reference

[S1] X. Chen, L. Guo, Q. Ma, W. Chen, W. Fan, J. Zhang, *Molecules* **2019**, *24*, 2950.

[S2] K. G. Brahmbhatt, N. Ahmed, S. Sabde, D. Mitra, I. P. Singh, K. K. Bhutani, *Bioorg. Med. Chem. Lett.* **2010**, *20*, 4416-4419.

[S3] L. Jiao, W. Pang, J. Zhou, Y. Wei, X. Mu, G. Bai, E. Hao, *J. Org. Chem.* **2011**, *76*, 9988-9996.

[S4] J. Liu, X. Liu, J. Qian, C. Meng, P. Zhu, J. Hang, Y. Wang, B. Xiong, X. Qiu, W. Zhu, Y. Yang, Y. Zhang, Y. Ling, *J. Med. Chem.* **2020**, *63*, 9271-9283.

[S5] Y. Liu, J. Zeng, Q. Li, M. Miao, Z. Song, M. Zhao, Q. Miao, M. Gao, *Advanced Optical Materials* **2022**, *10*, 2102709.

[S6] T. Meng, W. Ma, M. Fan, W. Tang, X. Duan, *Anal. Chem.* **2022**, *94*, 10109-10117.

[S7] H. Li, Q. Yao, W. Sun, K. Shao, Y. Lu, J. Chung, D. Kim, J. Fan, S. Long, J. Du, Y. Li, J. Wang, J. Yoon, X. Peng, *J. Am. Chem. Soc.* **2020**, *142*, 6381-6389.

[S8] X. Li, P. Wu, W. Cao, H. Xiong. *Chem. Commun.* **2021**, *57*, 10636-10639.
[S9] C. H. Tung, M. S. Han, Z. Shen, B. D. Gray, K. Y. Pak, J. Wang, *ACS Sens*. **2021**, *6*, 3657-3666.
[S10] H. Li, Q. Yao, F. Xu, N. Xu, W. Sun, S. Long, J. Du, J. Fan, J. Wang, X. Peng, *Front. Chem*. **2018**, *6*, 485.
[S11] H. Onoyama, M. Kamiya, Y. Kuriki, T. Komatsu, H. Abe, Y. Tsuji, K. Yagi, Y. Yamagata, S. Aikou, M. Nishida, K. Mori, H. Yamashita, M. Fujishiro, S. Nomura, N. Shimizu, M. Fukayama, K. Koike, Y. Urano, Y. Seto, *Sci. Rep*. **2016**, *6*, 26399.
[S12] T. Fujii, M. Kamiya, Y. Urano, *Bioconjug. Chem*. **2014**, *25*, 1838-1846.
[S13] Y. Urano, M. Sakabe, N. Kosaka, M. Ogawa, M. Mitsunaga, D. Asanuma, M. Kamiya, M. R. Young, T. Nagano, P. L. Choyke, H. Kobayashi, *Sci. Transl. Med*. **2011**, *3*, 110-119.
[S14] X. Wen, R. Zhang, Y. Hu, L. Wu, H. Bai, D. Song, Y. Wang, R. An, J. Weng, S. Zhang, R. Wang, L. Qiu, J. Lin, G. Gao, H. Liu, Z. Guo, D. Ye, *Nat. Commun*. **2023**, *14*, 800.
